# Faculty Training on Navigating Gender and Sex in Medical Education

**DOI:** 10.15766/mep_2374-8265.11427

**Published:** 2024-08-13

**Authors:** Benjamin Crosby, Hannah Dumas, Janet Monroe, Fredric Fabiano, Isabelle Gell-Levey, Christopher Noyes, Kikuye Sugiyama, Jennifer Siegel, Angelique Harris, Carl Streed, Ann C. Zumwalt

**Affiliations:** 1 Third-Year Medical Student, Boston University Chobanian & Avedisian School of Medicine; 2 Fourth-Year Medical Student, Boston University Chobanian & Avedisian School of Medicine; 3 Second-Year Medical Student, Boston University Chobanian & Avedisian School of Medicine; 4 Medical Director, Transgender Health Program, Massachusetts General Hospital; 5 Associate Dean, Office of Diversity and Inclusion, Boston University Chobanian & Avedisian School of Medicine; Associate Professor, Department of General Internal Medicine, Boston Medical Center; 6 Research Lead, GenderCare Center, Boston Medical Center; Assistant Professor, Section of General Internal Medicine, Boston University Chobanian & Avedisian School of Medicine; 7 Associate Professor, Department of Anatomy and Neurobiology, Boston University Chobanian & Avedisian School of Medicine; † Co-primary author

**Keywords:** Gender and Sexual Diversity, Sexual and Gender Minorities, Clinical Reasoning/Diagnostic Reasoning, Cultural Competence, Differences of Sexual Development, Faculty Development, Gender Identity, Health Equity, LGBTQ+, Diversity, Equity, Inclusion

## Abstract

**Introduction:**

Language that assumes gender and sex are binary and aligned is pervasive in medicine and is often used when teaching on physiology and pathology. Information presented through this lens oversimplifies disease mechanisms and poorly addresses the health of gender and sexually diverse (GSD) individuals. We developed a training session to help faculty reference gender and sex in a manner that would be accurate and inclusive of GSD health.

**Methods:**

The 1-hour session for undergraduate and graduate medical educators highlighted cisgender and binary biases in medical teachings and introduced a getting-to-the-root mindset that prioritized teaching the processes underlying differences in disease profiles among gender and sex subpopulations. The training consisted of 30 minutes of didactic teaching and 20 minutes of small-group discussion. Medical education faculty attended and self-reported knowledge and awareness before and after the training. Results were compared using paired *t* tests. Expenses included fees for consultation and catering.

**Results:**

Forty faculty participated (pretraining survey *n* = 36, posttraining survey *n* = 21). After the training, there was a significant increase in self-reported awareness of the difference between gender and sex (*p* = .002), perceived relevance of gender to teachings (*p* = .04), and readiness to discuss physiological drivers of sex-linked disease (*p* = .005).

**Discussion:**

Participants reported increased understanding and consideration of gender and sex in medical education; feedback emphasized a desire for continued guidance. This easily adaptable session can provide an introduction to a series of medical teachings on gender and sex.

## Educational Objectives

By the end of this session, faculty learners will be able to:

1. Describe the pervasiveness of cisgender and gender binary biases in medical education.
2. Appreciate how use of the terms *women/female* and *men/male* in medical training can exclude or hold ambiguous applicability for transgender and gender-diverse people, thereby warranting clarification and context.
3. Begin to describe and teach the pathophysiological factors that drive differences in disease profiles among populations that have previously been grouped into gender and sex categories in an undergraduate/graduate medical education setting.

## Introduction

Binary gender and sex categories are utilized throughout medical education to teach characteristics, processes, and disease patterns. The practice of teaching disease prevalence as linked to gender and sex (e.g., “Disease X is typically found in women/females ages 30–50”) implies that gender/sex is the risk factor for the disease, rather than more explicitly describing the gender-/sex-linked mechanisms that drive the disease patterns. This practice obscures the mechanisms that drive disease patterns and ultimately limits learners’ ability to interpret variations observed clinically (Table 1).

**Table 1.**
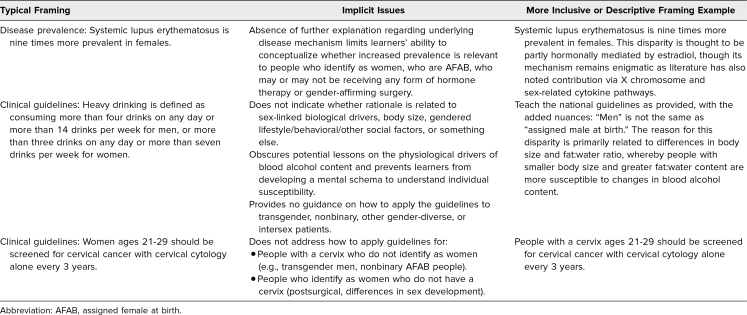
Getting to the Root of Gender and Sex Examples

Furthermore, many clinical guidelines and epidemiological patterns are framed in a manner that assumes all individuals’ sex assigned at birth and gender identity are congruent (cisgender) and strictly male or female (binary). These practices are problematic because these framings exclude gender-diverse people, such as those whose gender identity does not align with their sex assigned at birth, and people with differences of sex development or intersex characteristics (see Appendix A for definitions). Such framings essentially negate the existence of gender-diverse and intersex patients and inadequately prepare trainees to provide appropriate care for these patients. This concern is particularly poignant in light of extensive evidence that provider-driven stigma against and uninformed care for gender and sexually diverse (GSD) people is one of the the greatest barriers to care for GSD patients.^1–4^

### Getting to the Root of Gender and Sex

In recognition of the problems of cisgender bias described above, we developed a training session to guide faculty on how to more inclusively frame information related to gender or sex. The goals of this training are to demonstrate the pervasiveness of binary framings of gender and sex in medical education, explain why such framings limit learners’ understanding and exclude GSD people, and suggest alternative framings using typical teaching examples. Grounding medical teachings in lessons that explain the biological drivers (e.g., estrogen/testosterone levels, presence/absence of certain organs, chromosome expression, behavioral or environmental factors, etc.) enables educators to teach the mechanisms that underlie observed differences rather than using sex or gender categories as proxies for these mechanisms. This training is designed to help faculty reframe lessons to prioritize the mechanisms (the root) that drive differences between various gender and sex patient populations, rather than simply ascribing differences to binary gender/sex demographic categories. The dissection and intentional reframing of traditional cisnormative and binary teachings to physiology-rooted models serve the understanding of all patient health and, in particular, GSD populations.

## Methods

This investigation was deemed exempt by the Boston University Medical Campus Institutional Review Board (H-42554).

### Overview

This 1-hour session was intended for faculty teaching in any part of an undergraduate or graduate medical curriculum. It included a brief introduction to the history of GSD health care in the United States, an overview of the pervasiveness of cisnormative and binary categorizations of gender and sex in medical education, a dialogue on how the assumptions underlying these framings negate the existence of GSD identities, and a case presentation with facilitated small-group discussions. At our institution, the session was developed and presented by both faculty and students to demonstrate how both groups could benefit from this approach. We advertised this voluntary and stand-alone session on medical faculty email distribution lists. Expenses included lunch catering and a consultation fee, which may not be an additional expense for other groups that consider adapting this training without further consultation input. No incentive was provided for completion of pre- or postsurvey forms.

The training was designed to have the option to be delivered in person, remotely, or in a hybrid format, and facilitators could mediate either live or remote audiences. When delivered at our institution, participants attended both remotely and in person.

#### Prework: vocabulary

To save time during the workshop, we asked that participants review a video defining core terminology before the workshop.^5^ Specifically, this prework defined and described the differences between gender and sex; provided brief definitions of gender identity and pronoun usage, differences of sex development/intersex features, and sexual orientation; and offered examples across the spectrums of each category.

#### Part 1: didactic (Appendix B)

The didactic portion of the session was approximately 30 minutes long and consisted of three parts: (1) contextualization of the training in the current state of GSD health/health care in the United States, (2) illumination of problematic issues with the typical framings of sex and gender, and (3) description of a getting-to-the-root mindset.

At our institution, the training was presented by a consultant, faculty member, and medical student, although the training could be implemented by any combination of roles. The consultant was an expert on GSD health issues who also provided insights during the development of the session. In our training, the consultant introduced the session with a presentation on its relevance in the context of the history of GSD health care in the United States, with a focus on the evolution of the medical community's understanding of GSD health and evidence connecting GSD disparities with inadequate care within the medical system. These lessons were used to frame why it was incumbent upon current medical educators to improve the inclusivity of medical training. While this content was an important part of the training, it was not essential that it be presented by an external consultant or expert on GSD health.

A faculty member (author Ann C. Zumwalt) then reviewed fundamental vocabulary related to gender and sex and highlighted how these concepts appeared in medical education. A primary objective of this section was to demonstrate that not only were gender and sex ubiquitous in medical education but that they were typically presented without questioning the underlying assumptions of cisnormative or binary perspectives. This section also highlighted how gender and sex were frequently conflated and delineated the repercussions of these practices.

In the final component of the didactic section, a medical student (author Benjamin Crosby) introduced a getting-to-the-root approach to gender and sex, defining this approach and describing techniques to apply it, including avoiding using sex as a proxy, when and how to prioritize anatomy-first language, and avoiding conflations of gender and sex. Practical tips for educators to monitor and modify their practices during teaching were presented throughout this portion of the workshop.

#### Part 2: small-group session (Appendices C and D)

The workshop participants were then divided into small groups and worked through a 15-minute learning case. This portion of the training was designed for groups of six to eight people with two to three facilitators per group. At our institution, we formed three discussion groups, two in person and one remote via teleconference, each with approximately eight participants and facilitated by one faculty and two student team members.

Participants were provided with current national diagnosis guidelines for binge drinking and heavy alcohol use^6^ and a series of discussion prompts designed to foster a critical examination of the biases inherent to how gender and sex were referred to in the guidelines, groups considered, and ways to modify presentation of this information to be more inclusive (Appendix C).

Facilitators received a facilitator guide (Appendix D) detailing the key points to cover for each of the discussion questions to guide group conversation and ensure learning objectives were met in all groups. The facilitator guide also included hypothetical comments or questions that might arise, such as “What terminology can I use when presenting guidelines to not offend anyone?” and “I don't understand how I can build this into my teaching,” along with suggested responses.

#### Part 3: large-group discussion

The small groups reconvened after the breakout sessions to discuss and review what participants had learned. The workshop concluded with a summary of recommendations for modifying teaching practices to apply a getting-to-the-root approach to gender and sex, including techniques to address topics where the mechanism driving differences between gender and sex populations was unknown. Participants were provided with a handout outlining these recommendations and summarizing key takeaways from the session (Appendices E and F).

### Analysis

Before and after the training, participants were surveyed on self-reported knowledge of the difference between gender and sex, awareness of GSD health inequities, perceived relevance of sex and gender to course teachings, and comfort discussing factors underlying differences in disease incidence among different gender and sex groups (Appendix G). Respondents also indicated their current teaching responsibilities, prior experiences with GSD training, and optional demographic information regarding gender, race and ethnicity, and lesbian, gay, bisexual, transgender, queer/questioning (LGBTQ+) identity. Participants completed the online surveys using links provided before and at the closing of the session (Qualtrics XM). Surveys were labeled with a unique and anonymous participant-chosen identifier to allow matching of pre- and posttraining responses.

Survey responses were analyzed using Excel and SPSS 27 (IBM). Descriptive survey answers were scored on a 5-point Likert scale (1 = *not at all*, 2 = *slightly*, 3 = *moderately*, 4 = *very*, 5 = *extremely*) for analysis. We assessed distribution and variance patterns using normal Q-Q plots and *F* tests, which demonstrated normal distribution and equal variances in the datasets. Subsequent comparisons of pre- and postsurvey data were made using paired *t* tests with equal variance (α = .05, two-tailed) and paired power analyses (single power value = .80, two-tailed; Pearson product-moment correlation coefficient = .5).

## Results

The training session was attended by 40 faculty members. The pretraining survey was completed by 36 faculty (90% response rate), of whom 12 (33%) taught only in the preclerkship curriculum, four (11%) taught only in the clerkship curriculum, and 15 (42%) taught in both curricula. Five participants (14%) reported that they did not teach in either curriculum. The majority of respondents did not identify as LGBTQ+ (83%), and no respondent identified as nonbinary, though with the caveat that this was an optional demographic question that may have been skipped to avoid compromising anonymity. The majority of respondents had previous training (formal and/or informal) on GSD-related topics (89%; Table 2).

**Table 2.**
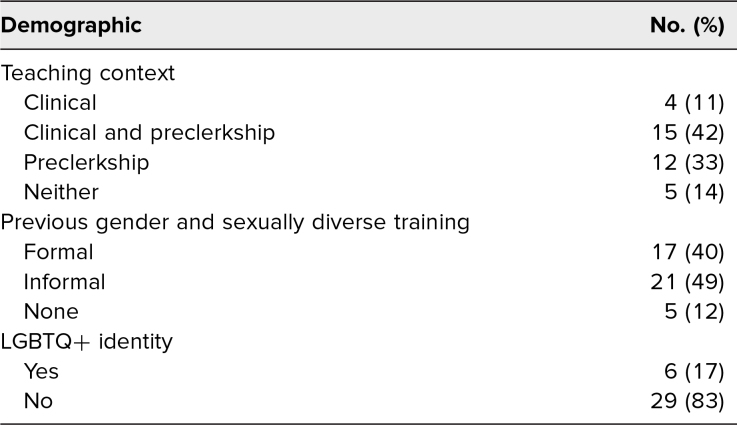
Faculty Training Audience Demographic Information (*N* = 36)

Prior to the training, most faculty self-reported that they felt moderately or very knowledgeable about the difference between gender and sex (48% and 33%, respectively; Table 3). While most faculty felt topics of sex were moderately, very, or extremely relevant to their teachings (43%, 24%, and 19%, respectively), faculty were more divided about the relevance of gender, with respondents indicating slight (29%), moderate (19%), very (33%), and extreme (14%) relevance. Before the training, half of the respondents reported that they felt moderately prepared to discuss factors contributing to differences in disease incidence for gender/sex biases (52%), while the majority of remaining respondents felt slightly (19%) or not at all (19%) prepared.

**Table 3.**
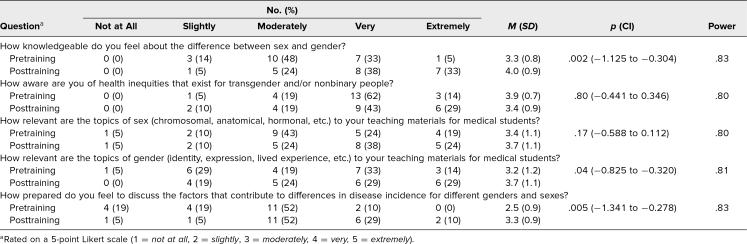
Pre- and Posttraining Results (*N* = 21)

The posttraining survey was completed by 21 faculty (52% response rate), of whom 15 (71%) had some preclerkship teaching responsibilities, 10 (48%) had some clerkship teaching responsibilities, and 18 (86%) did not identify as LGBTQ+. Normal Q-Q plot points for all five pre- and posttraining GSD comfort/awareness/knowledge-related questions fell on a 45-degree-angle reference line, so were deemed to follow a normal distribution. *F*-test values for these five questions (0.77, 0.59, 0.94, 1.13, and 1.03, respectively) were all below the *F* critical value of 2.23 (α = .05, *df* = 20) and regarded as demonstrating similar variability. Following the training, a significantly increased number of the 21 respondents who completed both pre- and posttraining surveys self-reported they were very or extremely aware of the difference between gender and sex (38% and 33%, respectively; *p* = .002). A significantly increased number of respondents self-reported that gender was moderately, very, or extremely pertinent to their teaching materials (24%, 29%, and 29%, respectively), and no participants after the session indicated that gender had no relevance (*p* = .04). Furthermore, a significantly increased number of participants reported feeling moderately, very, or extremely prepared to teach on the factors underlying differences in disease incidence among different gender and sex subgroups (52%, 29%, and 10%, respectively; *p* = .005; Table 3).

A majority of respondents (81%) reported after the training that they probably or definitely learned something that would change their teaching practice, and 95% indicated interest in attending similar training sessions in the future.

Participants’ feedback comments on the session are shown in Table 4.

**Table 4.**
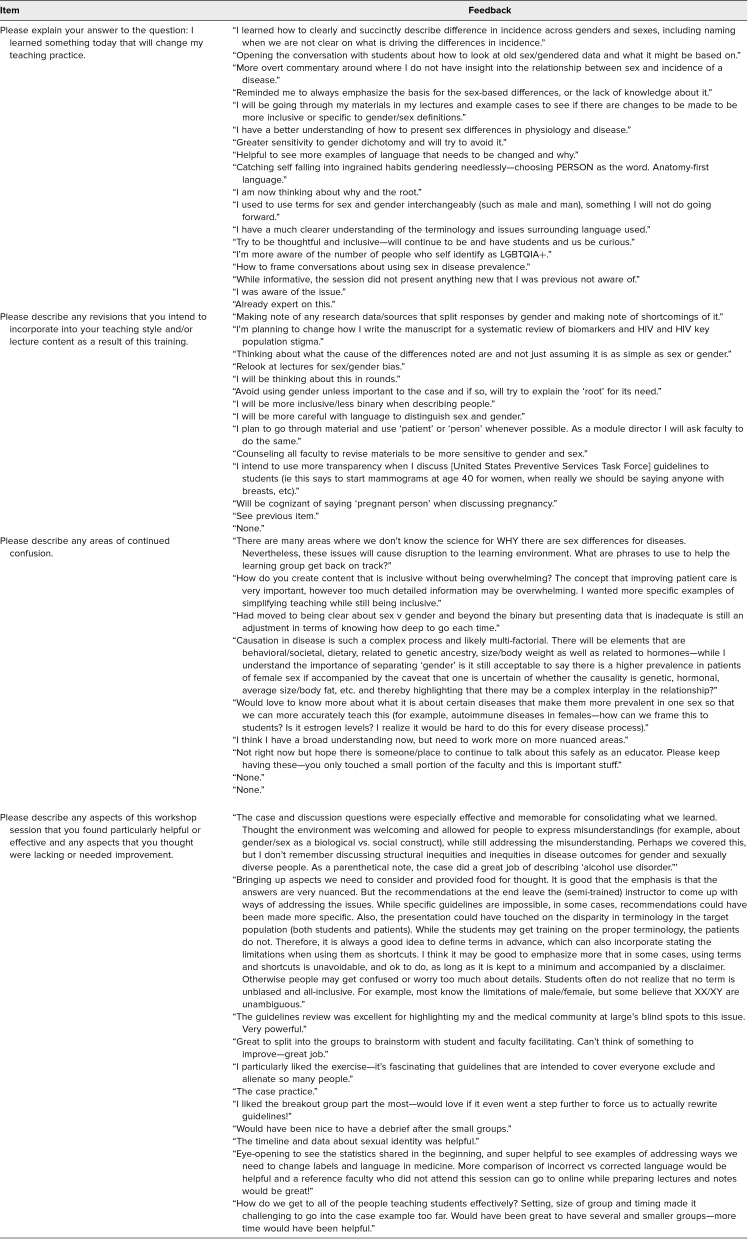
Posttraining Open-ended Feedback

## Discussion

Many published curricula and courses on topics related to diversity of gender or sexual orientation exist. Existing trainings address domains such as clinician understanding of GSD populations,^7^ terminology,^8^ and appropriate patient interactions.^9^ All are important for helping providers strengthen the therapeutic alliance with their GSD patients and deliver thoughtful care. The faculty training described here contributes to these efforts as it explores the pervasiveness of cisnormative and binary framings around gender and sex in medical education and why this is problematic, as well as offering the opportunity to develop techniques to avoid these biases.

This training was educationally impactful to an audience that predominantly had already participated in previous training on GSD topics. Two of the three teaching domains that demonstrated significant improvement in reported comfort and awareness after the session reflected the major teaching points of the session, namely, the practice of a getting-to-the-root mindset and the avoidance of conflating gender and sex. Participants also reported a significant increase in awareness of the relevance of gender to their respective teaching content.

Open-ended feedback on the session (Table 4) noted the effectiveness of the small-group discussion, as illustrated by a comment that mentioned how the deconstruction of the provided national guidelines was “excellent for highlighting my and the medical community at large's blind spots to this issue.” Literature on medical faculty development emphasizes the importance of training activities that facilitate reflection, interdisciplinary collaboration, and group discussion for the promotion of collegiality and creation of a learning community.^10^ We incorporated all three of these items into a single 1-hour session, and it is encouraging to appreciate the benefits from the synthesis of these elements as demonstrated by the majority of respondents indicating an intent to change teaching practices and as supported by specific posttraining reflections (Table 4).

It is important to call attention to the nature of the learning environment for this training. One respondent commented on the importance of safe spaces for educators to discuss without the inhibitory fear of making a mistake. Though the classroom environment is typically intended to be an opportunity for trial-and-error for students, faculty are often not afforded similar leeway to make mistakes. Feedback on this session alluded to a need for space for faculty to openly discuss in the process of evaluating and adjusting their lecture content, as should be afforded to all learners.

Participant feedback also highlighted opportunities to improve the training. Only 15 minutes were allotted for the small-group discussion, and feedback indicated that additional time would have been helpful (Table 4). If the overall session were extended in length (e.g., 90 minutes rather than 60 minutes), the training would benefit from more time afforded to the small-group discussion. Other feedback indicated that more specific recommendations for the implementation of a getting-to-the-root mindset would have been appreciated. This comment is mirrored by the finding that, though a statistically significant number of respondents indicated increased preparedness to utilize a getting-to-the-root approach after the session, the majority still felt only moderately prepared (Table 3). Ideally, additional sessions, variety and complexity of case examples, and opportunities for practice would better support faculty preparation.

While the content of this training session has been designed for both undergraduate and graduate medical educators, we only surveyed participants’ roles in the undergraduate medical curriculum. Future adaptations of this training session could consider specific outreach to graduate medical educators to understand its impact at later phases of medical education.

The overall findings from the session are particularly compelling as there is a paucity of prior literature on medical faculty training in teaching on gender and sex in a manner inclusive of GSD health. Given the supportive feedback and various strengths delineated in the survey results, the structure of the training session is effective and flexible and can be utilized to guide future training both within and beyond our institution. The training is also effective in that it is relatively short and inexpensive, as the session has been designed to last for a single lunch hour to accommodate audience availability.

There are important limitations to acknowledge regarding our interpretation of this training session and its impact. Only 36 participants completed the pretraining survey, and because many participants were unable to stay through the session due to professional responsibilities, only 21 posttraining survey responses were collected. Thus, despite reported power values of above 80% in all five questions, the small sample size still inherently limits the statistical significance of our results and our assuredness of the session's potential impact at other institutions. Furthermore, we were unable to rule out chance or to complete a sensitivity analysis of our results. A potential selection bias underlies the survey results as faculty self-selected to attend this optional training session and presumably had inherent interest in learning about GSD-related topics, so our results may not be wholly representative of overall faculty consensus. Lastly, pre- and posttraining survey questions were based on self-reported perceptions of knowledge, comfort, and awareness and did not objectively test respondent skills or knowledge.

This 1-hour training session introduces and guides medical educators to thoughtfully teach about gender and sex in a manner that is accurate and more inclusive of GSD health. Similar forums in the future with particular attention to case presentation examples on utilizing a getting-to-the-root approach to gender and sex will be helpful to continue supporting faculty learning.

## Appendices


Key Terms.docxPresentation With Speaker Notes.pptxSmall-Group Discussion Questions.docxFacilitator Guide.docxHandout Form (Printable Version, Trifold Format).pdfHandout Form (Electronic Version, Standard Format).pdfPre- and Posttraining Survey Forms.docx
*All appendices are peer reviewed as integral parts of the Original Publication.*
