# Supplementary material for: Faculty Training on Navigating Gender and Sex in Medical Education
Source: MedEdPORTAL. 2024 Aug 13;20:11427. doi: 10.15766/mep_2374-8265.11427 (PMC11319425; doi:10.15766/mep_2374-8265.11427)
Supplement: Supplementary file 1 — Key Terms.docxPresentation With Speaker Notes.pptxSmall-Group Discussion Questions.docxFacilitator Guide.docxHandout Form (Printable Version, Trifold Format).pdfHandout Form (Electronic Version, Standard Format).pdfPre- and Posttraining Survey Forms.docx [file mep_2374-8265.11427-s001.zip › F. Handout Form (Electronic Version, Standard Format).pdf]

Framework for Deconstructing Sex in Medical Education

THE GOAL OF THIS RESOURCE IS TO FACILITATE TRAINING STUDENTS IN A HOLISTIC MANNER THAT FOCUSES ON THE ROOT CAUSES OF SEX-RELATED DIFFERENCES IN MEDICINE

Sex is multi-faceted

Although often depicted as a binary of “male features” and “female features,” biological sex does not exist as a binary. Biological sex can refer to chromosomes, hormones, and/or anatomy, and there is variation in each. For example:

- Chromosomes can be XX, XO, XY, XXY, XXX [among others]
- People have varying amounts of and sensitivity to estrogen, androgen, testosterone and other sex hormones over the course of the lifespan. People can also take exogenous hormones and blockers for a variety of reasons.
- All anatomical features that are associated with sex can vary in size, phenotype, and presence/absence.
- As much as 2% of the population is born with phenotypic characteristics that fall outside of typical male/female. [1]

When using sex terms to describe biological traits, it is important to acknowledge the potential variation beyond the male/female binary.

Clarity and accuracy in language

When encountering binary language, pause and consider what you mean or what is meant by the writer.

- It can be appropriate to use male/female when describing a sex trait, but be careful not to assign identity/gender to these terms. E.g. Rather than “A fetus with XY chromosomes will be a male”, say “XY is defined as a male genotype.”
- It is appropriate to use gendered terms (man/woman/nonbinary) when the person has communicated their identity.
- When talking about groups of people, using male/female is often not complete or accurate. Consider who is being included and excluded in this framing. What population are you speaking about most specifically? For example, rather than “Females have an increase in blood volume when pregnant," try: “Pregnant people/ patients have increased blood volume."

Getting to the Root: Focus on what you mean

**INSTEAD OF**	<u>Women</u> should have pap testing every 3-5 years
**TRY**	<u>People with cervixes</u> should have pap testing every 3-5 years

Using language specific to 'cause-effect' physiological understandings and acknowledging complexities of the biological reality ensures a more accurate depiction of the biological topic being addressed.

Using binary sex terms without context or clarification often oversimplifies the intended lesson about mechanisms of human health, and excludes the health of intersex and transgender people. Instead, use anatomy-first language.

Questions to consider when preparing teaching materials

- Do sex or gender terms appear in your resources?
  - If so, make sure to fully clarify what these terms refer to.
  - e.g. does "female" refer to XX chromosomes, estrogen-driven physiology, people who ovulate, people with vulvas? Something else?
- When citing literature, do the resources you cite divide the information by gender and/or sex? If so, how are these categories defined? Can you as an educator further clarify the resources?
- Is the literature inclusive of transgender and intersex populations? If not, how can you address that when you present the material? Can you describe how the information may differ in trans/intersex people?

When writing case stems

- When sex terms are used in clinical vignettes, is it possible to further clarify what they mean?
  - e.g. does male/female refer to anatomy, identity, something else?
- Do not assign gender to anatomy. Instead, use anatomy-first framing.
  - e.g. instead of "For the abdominal pain differential for women, don't forget about ovarian torsion," try "For patients with ovaries, torsion can cause intense abdominal pain and should therefore be on the differential."
- Do include variations in gender identity and sexual orientation in case stems throughout the curriculum

When presenting data categorized by sex, acknowledge if the data is not inclusive

The following phrases may be helpful:

- "In [this dataset/disease process] the 'sex -based' difference...
  - could be caused by [physiologic mechanism; social construct]"
  - is observable in epidemiological studies but the cause is unknown."
- "This trend needs to be further studied in a gender-diverse population."
- "Sex is not a binary, but the medical field has historically studied within this dichotomy."
- "Medical research has a history of leaving out or exploiting marginalized populations. When research excludes people, we have a weaker understanding of their health, and they receive poorer care for it, perpetuating health inequities."

Thank you!

THIS HAS BEEN A TEAM EFFORT BY GENDER AND SEXUAL DIVERSITY VERTICAL INTEGRATION GROUP, CHOBANIAN & AVEDISIAN BOSTON UNIVERSITY SCHOOL OF MEDICINE

A Living Document

Like virtually all fields, nothing is stagnant!
We are learning and sharing new verbiage all the time as we progress towards an increasingly inclusive vocabulary.

We hope that when you encounter the terms male/female in your materials, that you pause and think about it...
What do you mean?

Notes from session

Citations

1. Blackless, Melanie, Anthony Charuvastra, Amanda Derryck, Anne Fausto-Sterling, Karl Lauzanne, and Ellen Lee. 2000. How Sexually Dimorphic Are We? Review and Synthesis. American Journal of Human Biology 12 (2): 151–166. https://doi.org/10.1002/(SICI)1520-6300(200003/04)12:2%3c151::AID-AJHB1%3e3.0.CO;2-F.
