# Supplementary material for: Faculty Training on Navigating Gender and Sex in Medical Education
Source: MedEdPORTAL. 2024 Aug 13;20:11427. doi: 10.15766/mep_2374-8265.11427 (PMC11319425; doi:10.15766/mep_2374-8265.11427)
Supplement: Supplementary file 1 — Key Terms.docxPresentation With Speaker Notes.pptxSmall-Group Discussion Questions.docxFacilitator Guide.docxHandout Form (Printable Version, Trifold Format).pdfHandout Form (Electronic Version, Standard Format).pdfPre- and Posttraining Survey Forms.docx [file mep_2374-8265.11427-s001.zip › G. Pre- and Posttraining Survey Forms.docx]

**Appendix G. Pre- and post-training survey forms (approximately 5 minutes, each).** Survey forms can be distributed prior to and after the training session to help assess self-reported knowledge, comfort, and awareness regarding content from the teaching session.

**Study Consent**

This survey accompanies a workshop for Boston University Chobanian & Avedisian School of Medicine faculty on the integration of gender and sexually diverse (GSD) health in medical school teachings. This survey assesses knowledge and comfort with the topics addressed in this workshop. Please complete the survey to help us improve the quality of our presentations.

We are conducting a study on the effectiveness of this workshop on improving faculty comfort with navigating sex and gender in teaching. You may complete the survey without participating in the research.

If you agree to your information being included in the research study, please click “Yes” below.

If you complete the survey for QI purposes but do not wish to participate in the study, please click “No” below.

I agree to participate in this research (Y/N)

Risks to participating in the research study: Minimal

Survey questions address personal awareness and knowledge of sexual and gender minority topics, as well as optional demographic questions. There is a chance that participants may experience negative emotions related to these questions. Participants can halt participation in the study at any point or omit answers to individual questions without any impact on participation in the workshop session.

We will use participant-generated unique identifiers to track participation, and will not be collecting personally identifiable information. The anonymous data collected in this study will be stored on a spreadsheet only available to the study team, and will be destroyed upon completion of the study. Though we will make every effort to keep this data private, there is a chance of data breach resulting in release of participant responses. No personally identifiable information is being collected.

There is no compensation for participation in this study. The pre-survey is expected to take 5 minutes to complete and the post-survey is expected to take 10 minutes to complete. You may contact the Principal Investigator of this study at any time at azumwalt@bu.edu.

**Pre-Survey:**

Unique identifier: Please select a 5 character identifier for yourself that you will be able to remember using the following code: Number of siblings + first letter of middle name + first 3 letters of birth month (e.g. 2JApr). Please use a code that you will be able to remember as we will use these codes to compare pre- and post-survey responses. In order to maintain anonymity, please do not use your name or other easily identifiable information.

Code: _______

Demographic information

1. Do you have teaching responsibilities to preclinical students (M1 or M2)? (yes/no)
2. Do you have teaching responsibilities to clinical medical students (M3 or M4)? (yes/no)
3. Prior to this workshop, had you had any training (formal or informal) on topics of gender and sexual diversity? (formal, informal, other)
4. Do you identify as LGBTQ+? (*optional*)
5. What is your gender? (*optional*)

Survey questions

1. How knowledgeable do you feel about the differences between sex and gender?

1 2 3 4 5

not at all slightly moderately very extremely

1. How aware are you of health inequities that exist for transgender or non-binary individuals?

1 2 3 4 5

not at all slightly moderately very extremely

1. How relevant are the topics of sex (chromosomal, anatomical, hormonal, etc.) to your lecture material?

1 2 3 4 5

not at all slightly moderately very extremely

1. How relevant are the topics of gender (identity, expression, lived experience) to your lecture material?

1 2 3 4 5

not at all slightly moderately very extremely

1. How comfortable and prepared would you feel to elaborate on the various factors (e.g. genetic, hormonal, social) that contribute to differences in disease incidence between different sex- and gender-based demographics? (E.g. “males have higher rates of heart disease” vs “people with less estrogen hormone have a higher risk of heart disease because estrogen hormone is protective”)

1 2 3 4 5

not at all slightly moderately very extremely

**Post-Survey:**

Unique identifier: Please use the same 5 character identifier for yourself that you used in the pre-survey using the following code: Number of siblings + first letter of middle name + first 3 letters of birth month (e.g. 2JApr). In order to maintain anonymity, please do not use your name or other easily identifiable information.

Code: _______

1. How knowledgeable do you feel about the differences between sex and gender?

1 2 3 4 5

not at all slightly moderately very extremely

1. How relevant are the topics of sex (chromosomal, anatomical, hormonal, etc.) to your lecture material?

1 2 3 4 5

not at all slightly moderately very extremely

1. How relevant are the topics of gender (identity, expression, lived experience) to your lecture material?

1 2 3 4 5

not at all slightly moderately very extremely

1. How aware are you of health inequities that exist for transgender or non-binary individuals?

1 2 3 4 5

not at all slightly moderately very extremely

1. How comfortable and prepared do you feel to elaborate on the various factors (e.g. genetic, hormonal, social) that contribute to differences in disease incidence between different sex- and gender-based demographics? (E.g. “males have higher rates of heart disease” vs “people with less estrogen hormone have a higher risk of heart disease because estrogen hormone is protective”)

1 2 3 4 5

not at all slightly moderately very extremely

1. I learned something today that will change my teaching practice

1 2 3 4 5

no probably not unsure probably yes

1. Please explain your answer above
2. Please describe any revisions that you intend to incorporate into your teaching style and/or lecture content as a result of this training
3. Please describe any areas of continued confusion
4. Please describe any aspects of this workshop session that you found particularly helpful or effective, and any aspects that you were thought were lacking or needed improvement
5. If available, I would like to attend similar trainings in the future
