# Supplementary material for: Faculty Training on Navigating Gender and Sex in Medical Education
Source: MedEdPORTAL. 2024 Aug 13;20:11427. doi: 10.15766/mep_2374-8265.11427 (PMC11319425; doi:10.15766/mep_2374-8265.11427)
Supplement: Supplementary file 1 — Key Terms.docxPresentation With Speaker Notes.pptxSmall-Group Discussion Questions.docxFacilitator Guide.docxHandout Form (Printable Version, Trifold Format).pdfHandout Form (Electronic Version, Standard Format).pdfPre- and Posttraining Survey Forms.docx [file mep_2374-8265.11427-s001.zip › A. Key Terms.docx]

**Appendix A. Key Terminology (approximately 10 minutes).** Definitions for various key gender and sexually diverse-related terminology, several of which are included in the faculty training presentation, are included below for context and review.

LGBTQIA2S+: An abbreviation that stands for Lesbian, Gay, Bisexual, Transgender, Queer, Intersex, Asexual, and Two Spirit. LGBT+ is often used interchangeably, for brevity.

GSD: An abbreviation that stands for Gender and Sexual Diversity

Gender: Gender refers to the socially constructed categories that give rise to masculinity and femininity, and exists on a spectrum.^1^ Gender identity refers to a person’s own internal conception of their gender and it may evolve over time.^2^ Gender expression refers to signals they might put into the world that signify gender, which include ways of acting, dressing, etc.^2^ Gender is unrelated to sexual attraction or behavior.^3^

Non-binary Gender: A gender identity that does not align with the man-woman dichotomy.^3^

Gender Diverse: An umbrella term that includes non-cisgender gender identities

Gender expansive: A term for people who identify outside of traditional gender roles but do not feel tied to any one gender label.^4^

Two-Spirit: An exclusively indigenous gender identity, one who identifies with both masculine and feminine gender.^3^

Gender fluid: A gender that does not align with either end of the spectrum of gender, that may change and vary over time.^3^

Transgender: People who experience a discordance between their internal sense of gender and their sex assigned at birth.^2,3^

Cisgender: People whose gender identity is concordant with their sex assigned at birth.^3^

Genetic Sex: Humans have 23 sets of chromosomes, 22 autologous and 1 pair of sex chromosomes.^5^ A pair including XY is denoted genetically male, while a pair of XX denotes a female genetic sex. ^5^ People can have aneuploidy of sex hormones, most common including Turner syndrome (XO), Klinefelter syndrome (XXY), trisomy X (XXX), XYY, and XXYY. ^2,5^

Anatomic sex: Internal reproductive organs and external genitalia that a person may possess. Internal and external organs may be concordant (i.e. vulva externally with uterus and ovaries internally) or may not. For example, someone with turner syndrome may have vulva without a uterus.^7^ Sex assigned at birth is often based on what external genitalia a healthcare provider observes at the time of delivery.^8^

Intersex: A broad term used to describe individuals whose genetics, hormones, and/or anatomy does not align with strictly male or female.^3^

Estrogen driven physiology: Body development or physiology based on the actions of estrogens on various organ systems. Physiology that is mainly driven by estrogen may include growth of breast tissue, for example.^9^ Notably, people of all bodies and genotypes have both estrogen and testosterone, to varying levels.^10^

Testosterone driven physiology: Body development based on the actions of testosterone or other androgens on various organ systems. Physiology that is mainly driven by testosterone may include coarse facial hair development or voice deepening.^9^ Notably, people of all bodies and genotypes have both estrogen and testosterone, to varying levels.^10^

Queer: A term that was reclaimed by some to describe any non-cisgender or non-heterosexual identity. Also can be used as an umbrella term to describe the LGBT+ community.^11^

Lesbian: A person who identifies as a woman who experiences attraction to other women^3,11^

Gay: Sexual attraction to people of one’s own gender. ^3,11^

Bisexual: Sexual attraction to people of one’s own gender as well as other genders, or attraction regardless of gender. ^3,11^

Asexual: Someone who does not feel sexual attraction or desired partnered sexual activity.^3,11^

References

1. Short SE, Yang YC, Jenkins TM. Sex, gender, genetics, and health. Am J Public Health. 2013;103 Suppl 1(Suppl 1):S93-S101. doi:10.2105/AJPH.2013.301229
2. Teaching Gender Identity and Transgender Health with Jamie Feldman. Association of American Medical Colleges AAMC Videos and Resources. Washington, DC: October 2015. Accessed Feb 18, 2020. /initiatives/diversity/441824/teachingtransfeldman.html
3. Osmosis from Elsevier. Sexual Orientation and Gender Identity. Youtube. June 18, 2020. [Accessed August 3, 2023. https://www.youtube.com/watch?v=xCMmZUu07IQ](https://www.youtube.com/watch?v=xCMmZUu07IQ)
4. Resources on Gender-Expansive Children and Youth. Human Rights Campaign. Accessed February 19, 2024. https://www.hrc.org/resources/resources-on-gender-expansive-children-and-youth
5. Reale C, Invernizzi F, Panteghini C, Garavaglia B. Genetics, sex, and gender. J Neurosci Res. 2023;101(5):553-562. doi:10.1002/jnr.24945
6. Skuse D, Printzlau F, Wolstencroft J. Sex chromosome aneuploidies. Handb Clin Neurol. 2018;147:355-376. doi:10.1016/B978-0-444-63233-3.00024-5
7. Elamparidhi P, Kumar RR, Sivaranjinie S, Sibhithran R. Mullerian Agenesis Associated with Turner's Syndrome. J Clin Diagn Res. 2017;11(2):TD01-TD02. doi:10.7860/JCDR/2017/23305.9157
8. Burns E, Segaloff A, Carrera GM. Reassignment of sex: report of 3 cases. J Urol. 1960;84:126-133. doi:10.1016/S0022-5347(17)65502-2
9. Unger CA. Hormone therapy for transgender patients. Transl Androl Urol. 2016;5(6):877-884. doi:10.21037/tau.2016.09.04
10. Hammes SR, Levin ER. Impact of estrogens in males and androgens in females. J Clin Invest. 2019;129(5):1818-1826. doi:10.1172/JCI125755
11. General definitions. LGBT Resource Center. Accessed February 19, 2024. https://lgbt.ucsf.edu/glossary-terms.
