# Supplementary material for: Faculty Training on Navigating Gender and Sex in Medical Education
Source: MedEdPORTAL. 2024 Aug 13;20:11427. doi: 10.15766/mep_2374-8265.11427 (PMC11319425; doi:10.15766/mep_2374-8265.11427)
Supplement: Supplementary file 1 — Key Terms.docxPresentation With Speaker Notes.pptxSmall-Group Discussion Questions.docxFacilitator Guide.docxHandout Form (Printable Version, Trifold Format).pdfHandout Form (Electronic Version, Standard Format).pdfPre- and Posttraining Survey Forms.docx [file mep_2374-8265.11427-s001.zip › B. Presentation With Speaker Notes.pptx]

## Slide 1
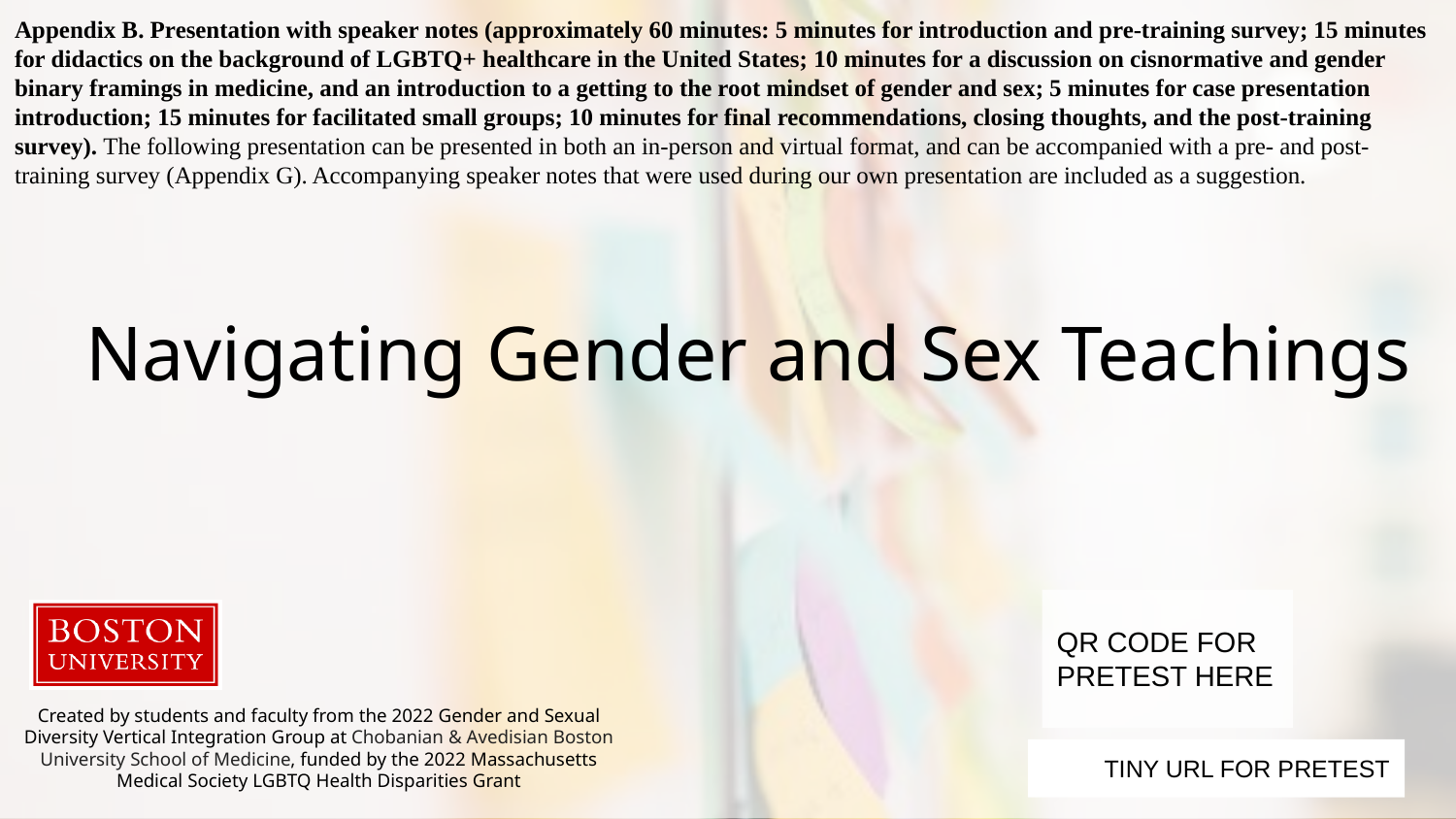

Appendix B. Presentation with speaker notes (approximately 60 minutes: 5 minutes for introduction and pre-training survey; 15 minutes for didactics on the background of LGBTQ+ healthcare in the United States; 10 minutes for a discussion on cisnormative and gender binary framings in medicine, and an introduction to a getting to the root mindset of gender and sex; 5 minutes for case presentation introduction; 15 minutes for facilitated small groups; 10 minutes for final recommendations, closing thoughts, and the post-training survey). The following presentation can be presented in both an in-person and virtual format, and can be accompanied with a pre- and post-training survey (Appendix G). Accompanying speaker notes that were used during our own presentation are included as a suggestion.
# Navigating Gender and Sex Teachings
QR CODE FOR PRETEST HERE
Created by students and faculty from the 2022 Gender and Sexual Diversity Vertical Integration Group at Chobanian & Avedisian Boston University School of Medicine, funded by the 2022 Massachusetts Medical Society LGBTQ Health Disparities Grant
TINY URL FOR PRETEST

## Slide 2
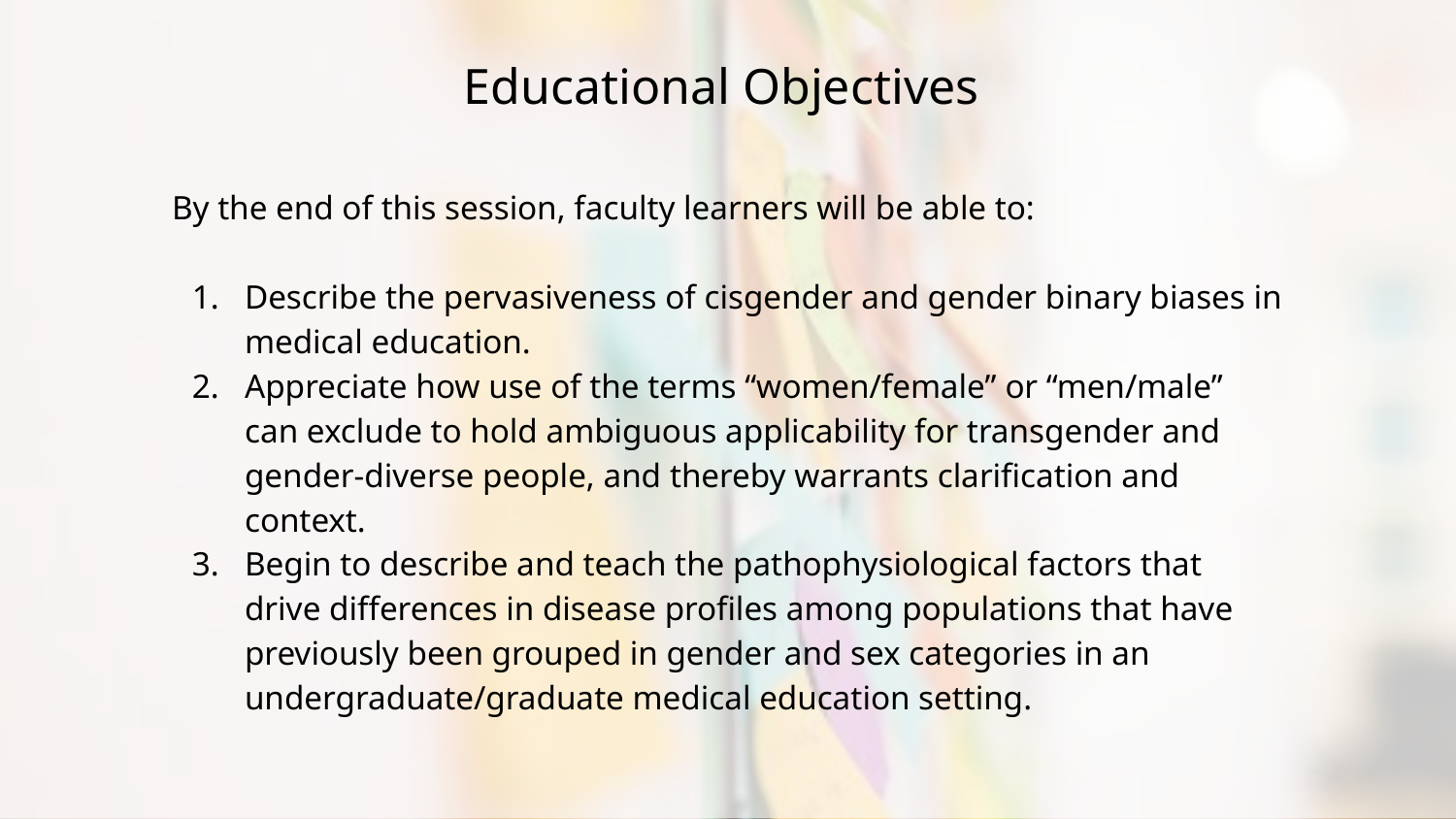

Educational Objectives
By the end of this session, faculty learners will be able to:
Describe the pervasiveness of cisgender and gender binary biases in medical education.
Appreciate how use of the terms “women/female” or “men/male” can exclude to hold ambiguous applicability for transgender and gender-diverse people, and thereby warrants clarification and context.
Begin to describe and teach the pathophysiological factors that drive differences in disease profiles among populations that have previously been grouped in gender and sex categories in an undergraduate/graduate medical education setting.

## Slide 3
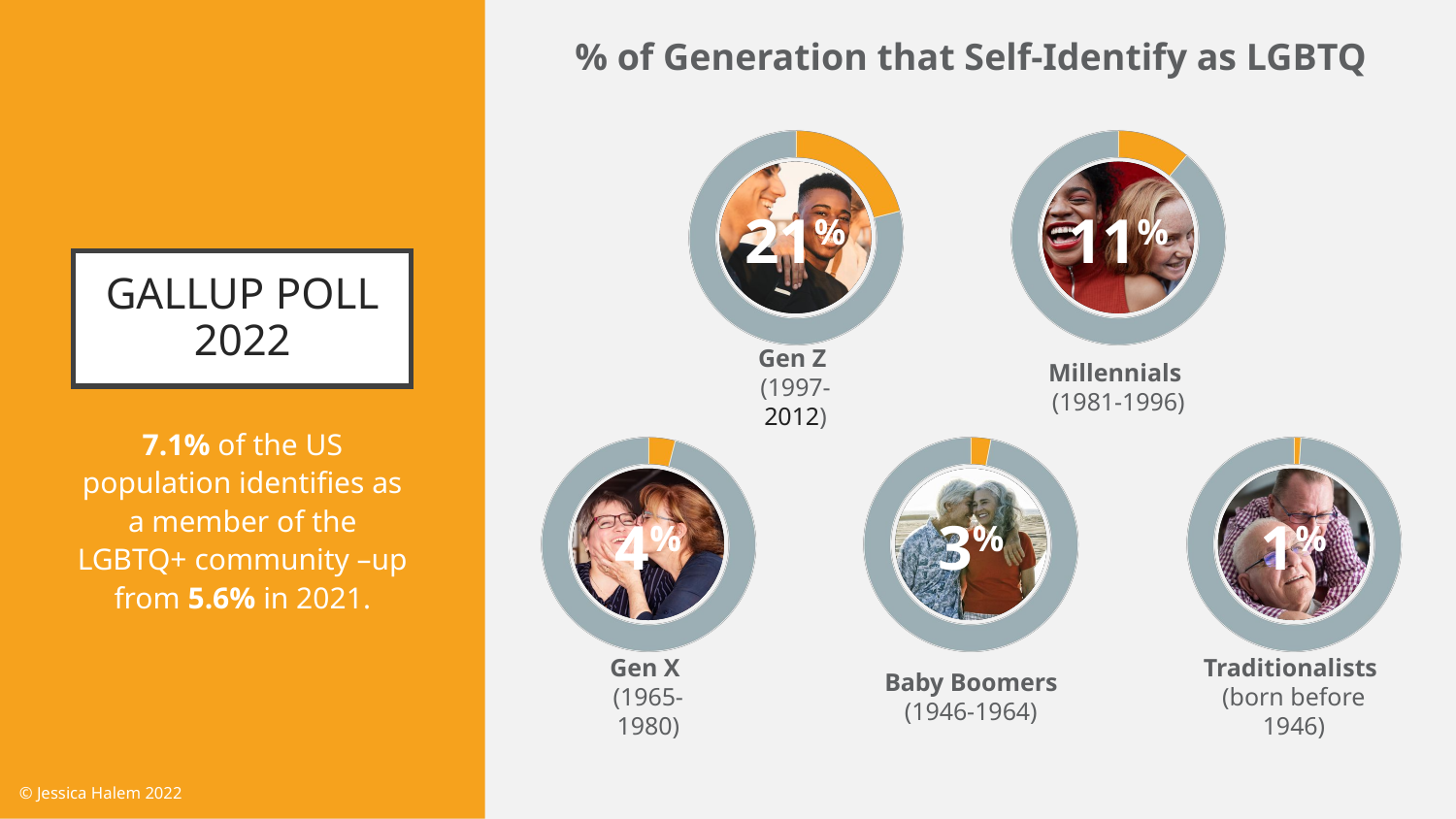

.
% of Generation that Self-Identify as LGBTQ
21%
11%
# GALLUP POLL 2022
Gen Z
(1997-2012)
Millennials
(1981-1996)
7.1% of the US population identifies as a member of the LGBTQ+ community –up from 5.6% in 2021.
4%
3%
1%
Gen X
(1965-1980)
Baby Boomers
(1946-1964)
Traditionalists
(born before 1946)
© Jessica Halem 2022

## Slide 4
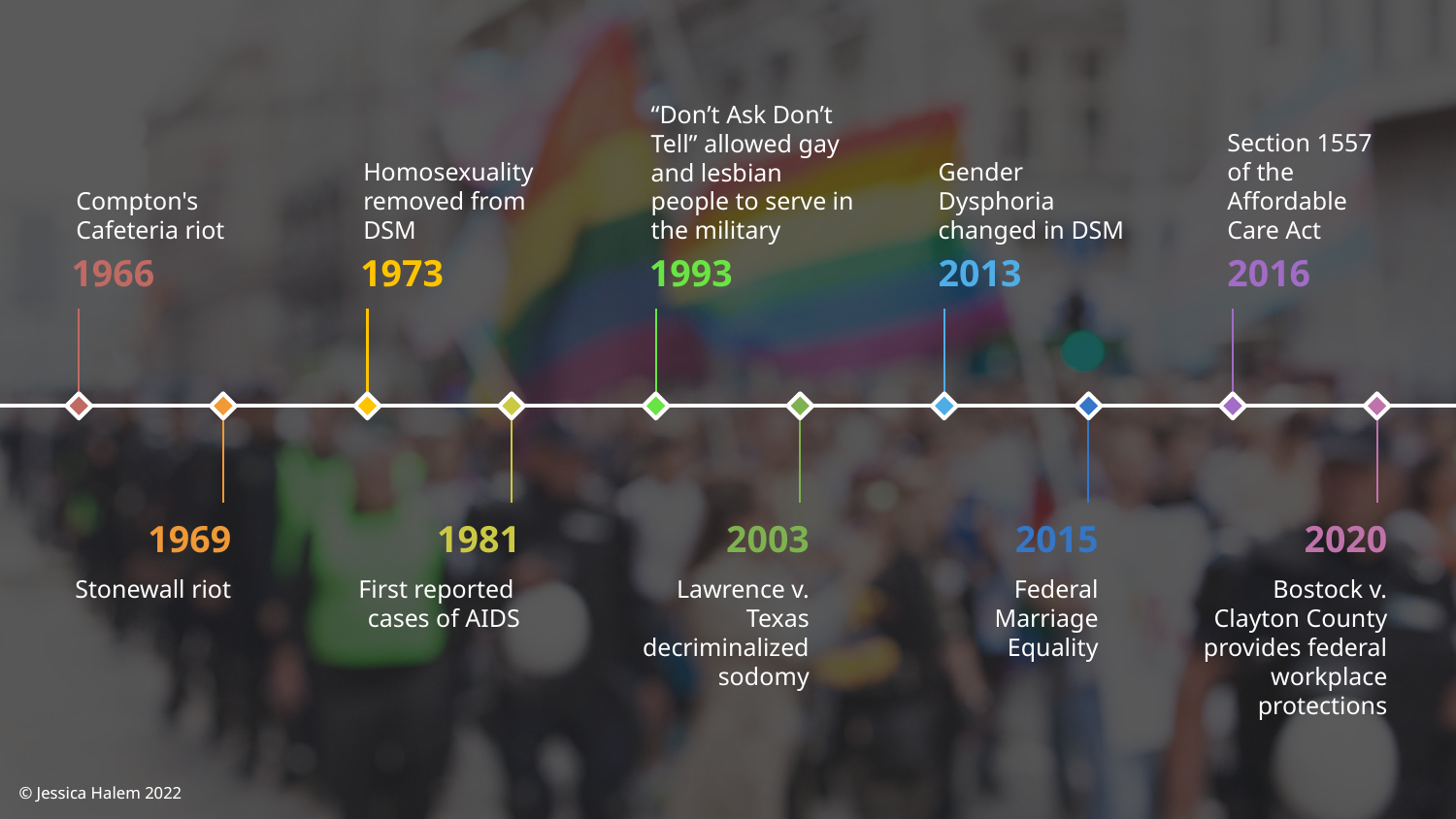

“Don’t Ask Don’t Tell” allowed gay and lesbian people to serve in the military
Section 1557 of the Affordable Care Act
Compton's
Cafeteria riot
Homosexuality removed from DSM
Gender Dysphoria changed in DSM
1966
1973
1993
2013
2016
1969
1981
2003
2015
2020
Stonewall riot
First reported
cases of AIDS
Lawrence v. Texas decriminalized sodomy
Federal Marriage Equality
Bostock v. Clayton County provides federal workplace protections
© Jessica Halem 2022

## Slide 5
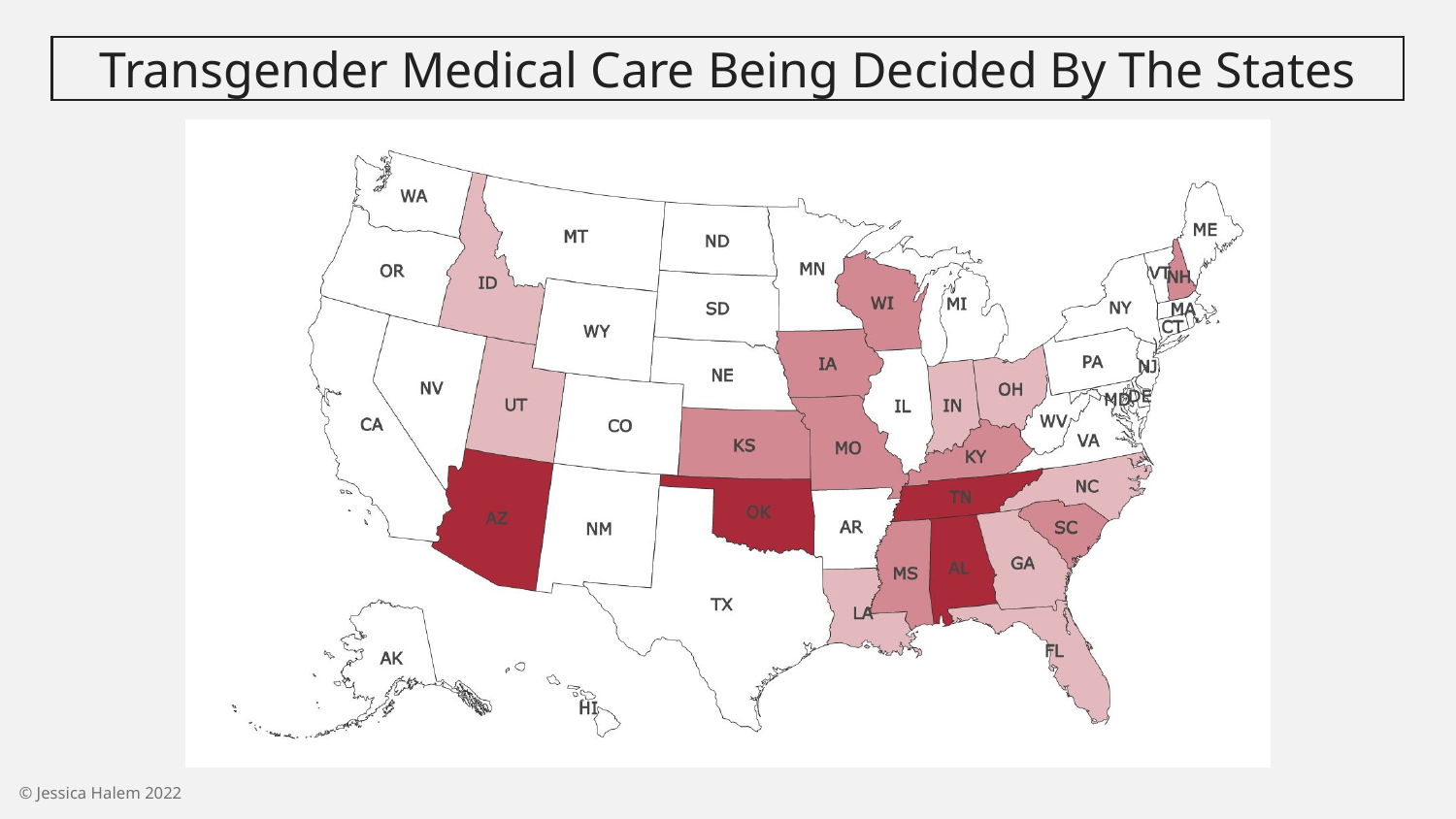

Transgender Medical Care Being Decided By The States

## Slide 6
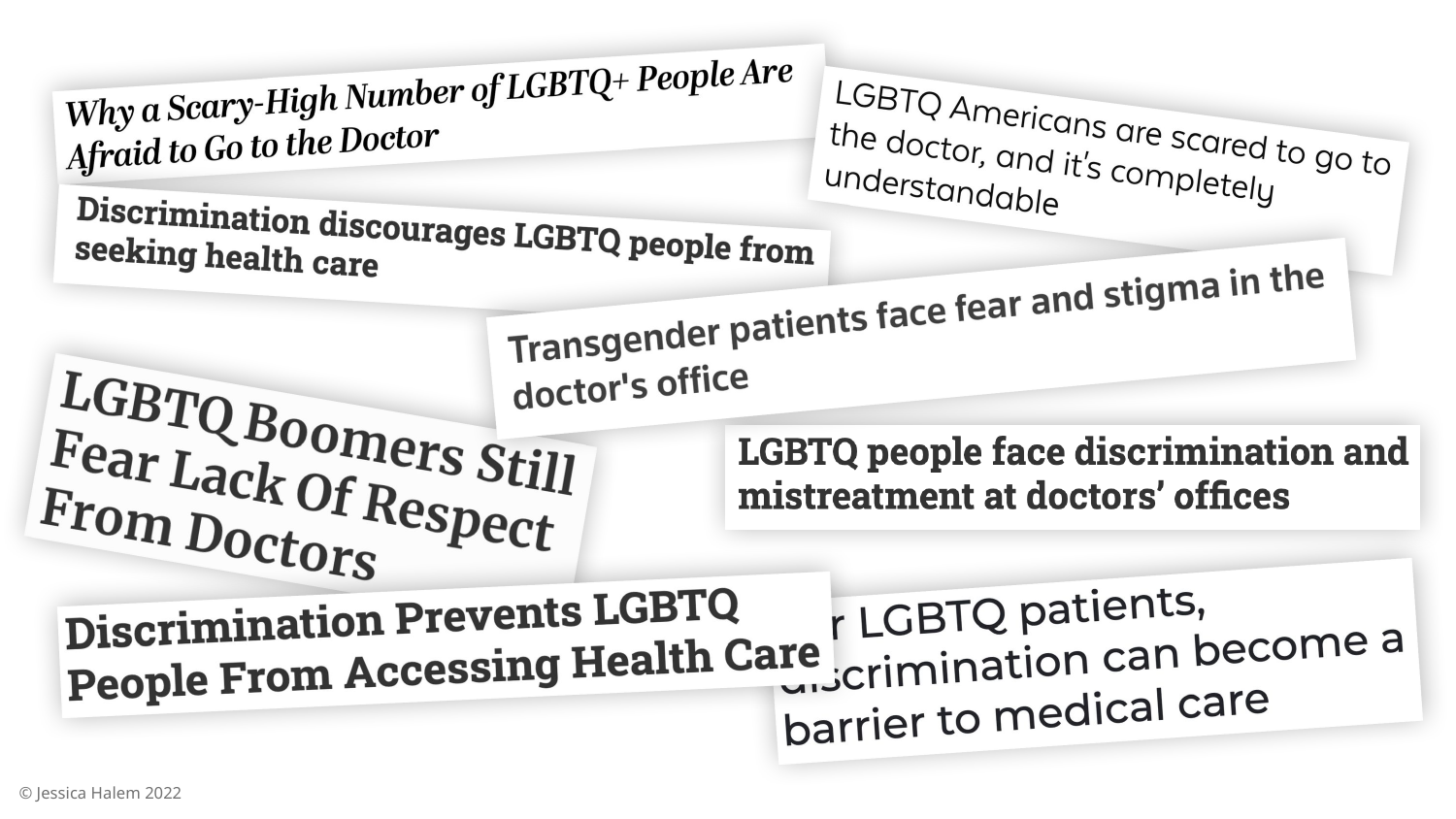

## Slide 7
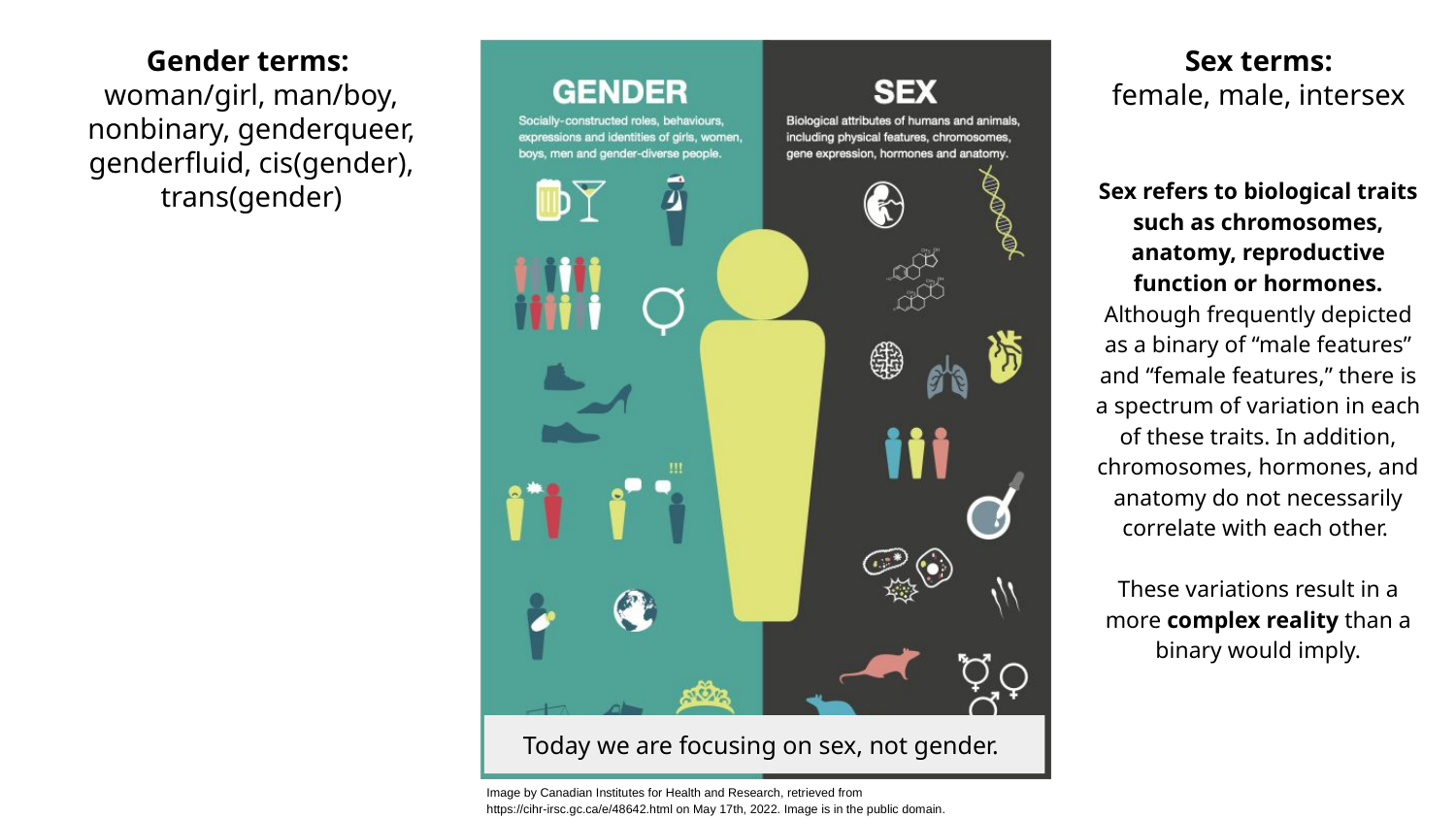

Gender terms:
woman/girl, man/boy, nonbinary, genderqueer, genderfluid, cis(gender), trans(gender)
Sex terms:
female, male, intersex
Sex refers to biological traits such as chromosomes, anatomy, reproductive function or hormones.
Although frequently depicted as a binary of “male features” and “female features,” there is a spectrum of variation in each of these traits. In addition, chromosomes, hormones, and anatomy do not necessarily correlate with each other.
These variations result in a more complex reality than a binary would imply.
Today we are focusing on sex, not gender.
Image by Canadian Institutes for Health and Research, retrieved from https://cihr-irsc.gc.ca/e/48642.html on May 17th, 2022. Image is in the public domain.

## Slide 8
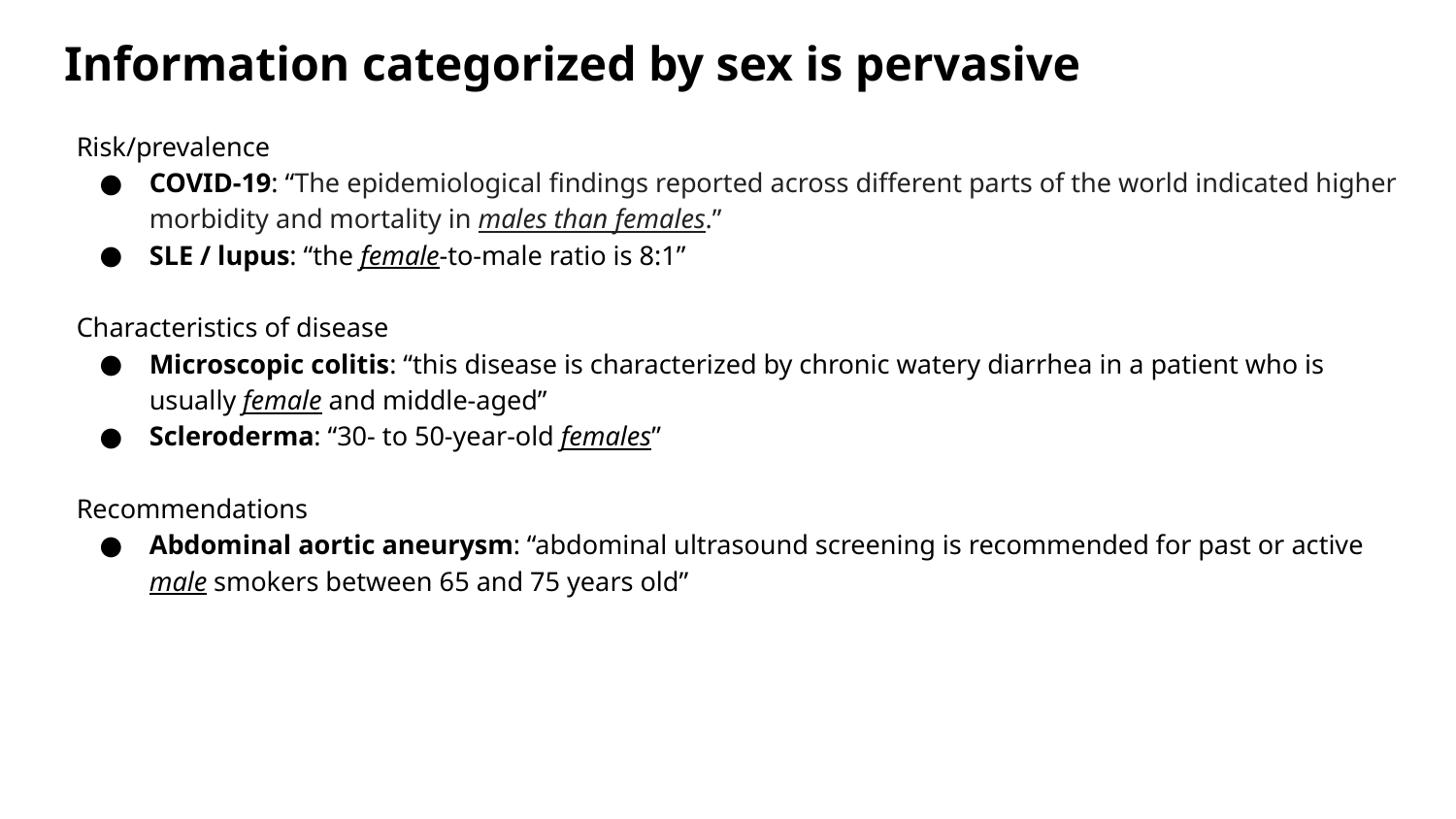

# Information categorized by sex is pervasive
Risk/prevalence
COVID-19: “The epidemiological findings reported across different parts of the world indicated higher morbidity and mortality in males than females.”
SLE / lupus: “the female-to-male ratio is 8:1”
Characteristics of disease
Microscopic colitis: “this disease is characterized by chronic watery diarrhea in a patient who is usually female and middle-aged”
Scleroderma: “30- to 50-year-old females”
Recommendations
Abdominal aortic aneurysm: “abdominal ultrasound screening is recommended for past or active male smokers between 65 and 75 years old”

## Slide 9
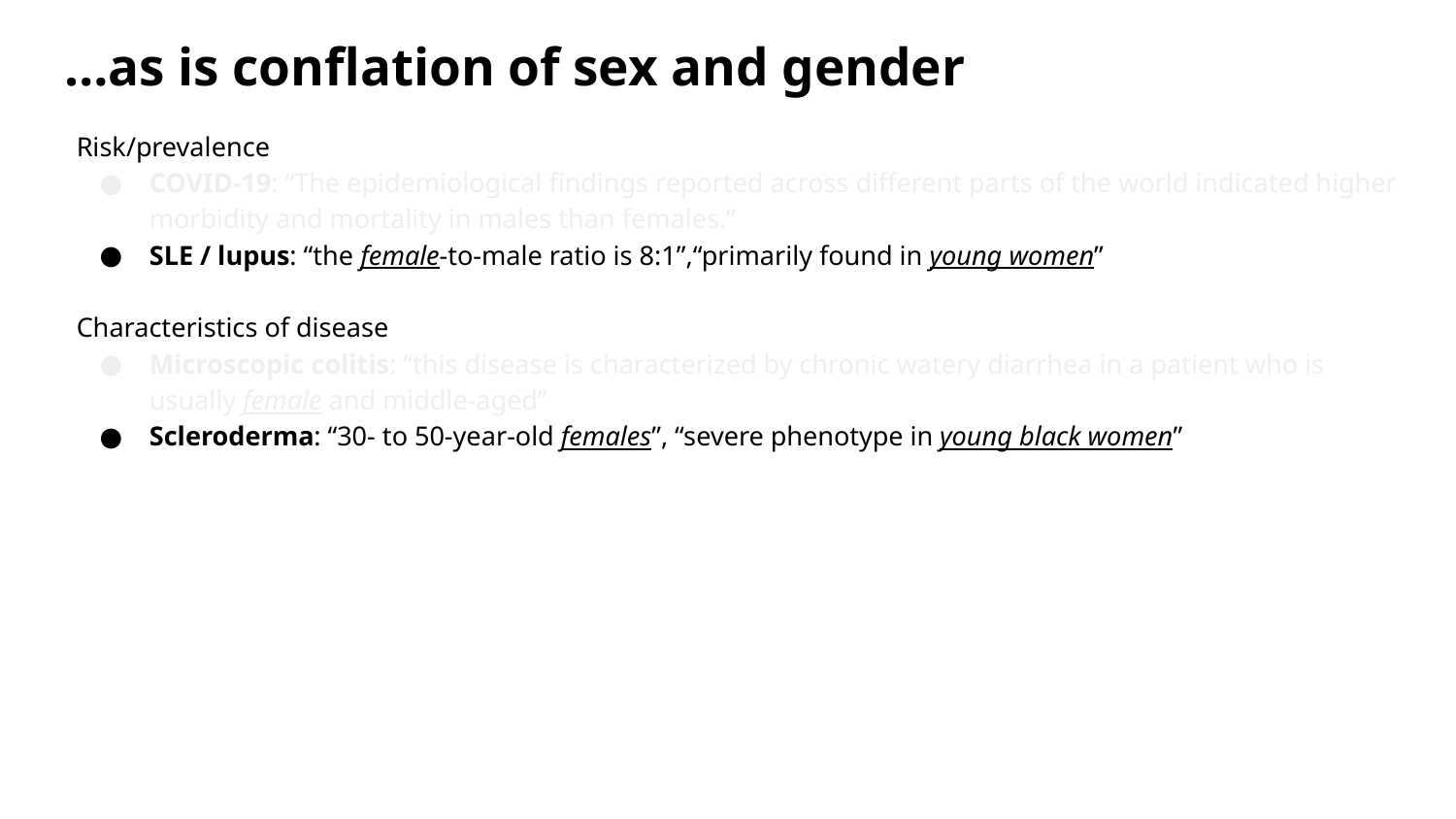

# …as is conflation of sex and gender
Risk/prevalence
COVID-19: “The epidemiological findings reported across different parts of the world indicated higher morbidity and mortality in males than females.”
SLE / lupus: “the female-to-male ratio is 8:1”,“primarily found in young women”
Characteristics of disease
Microscopic colitis: “this disease is characterized by chronic watery diarrhea in a patient who is usually female and middle-aged”
Scleroderma: “30- to 50-year-old females”, “severe phenotype in young black women”

## Slide 10
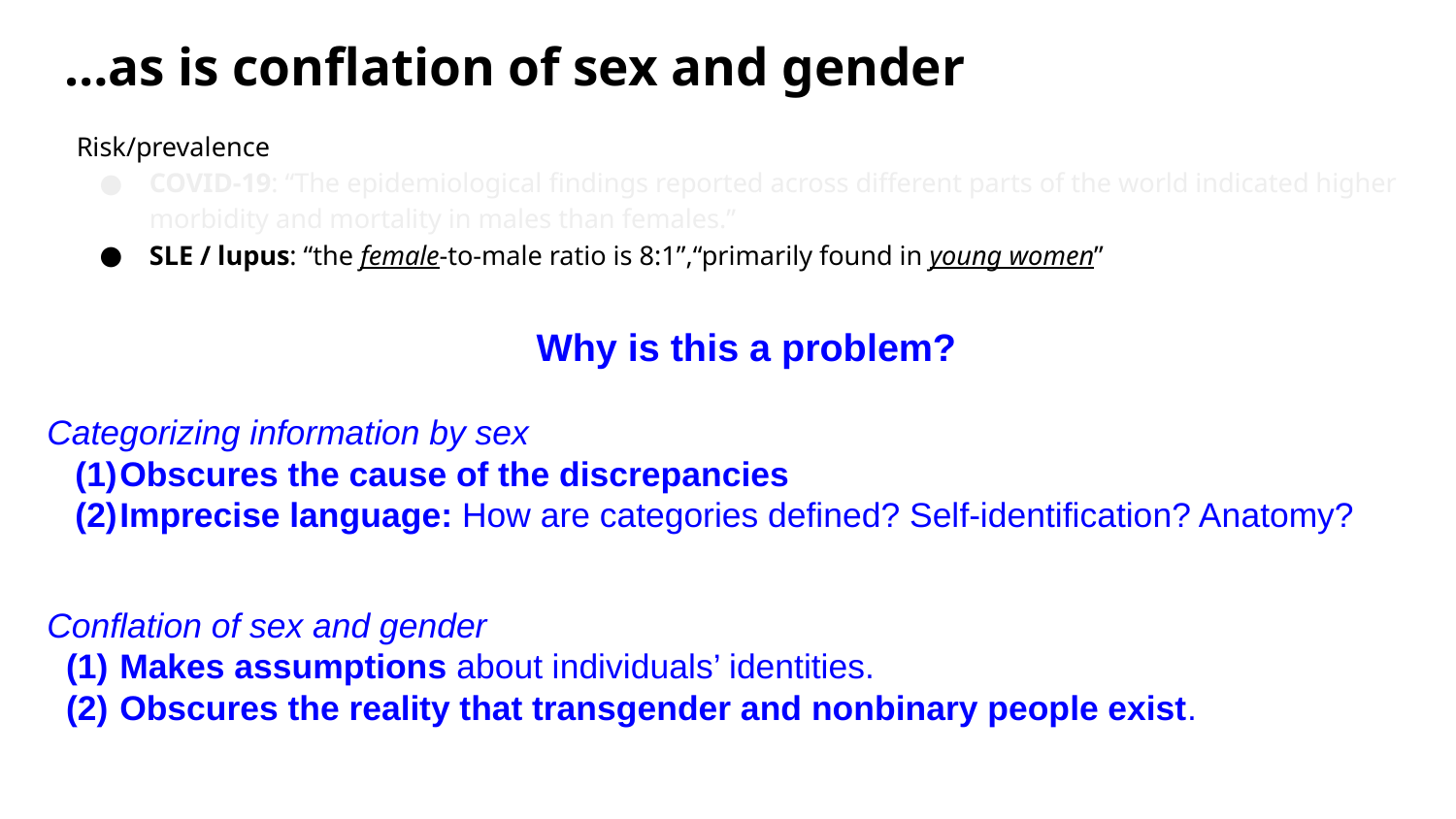

# …as is conflation of sex and gender
Risk/prevalence
COVID-19: “The epidemiological findings reported across different parts of the world indicated higher morbidity and mortality in males than females.”
SLE / lupus: “the female-to-male ratio is 8:1”,“primarily found in young women”
Why is this a problem?
Categorizing information by sex
Obscures the cause of the discrepancies
Imprecise language: How are categories defined? Self-identification? Anatomy?
Conflation of sex and gender
Makes assumptions about individuals’ identities.
Obscures the reality that transgender and nonbinary people exist.

## Slide 11
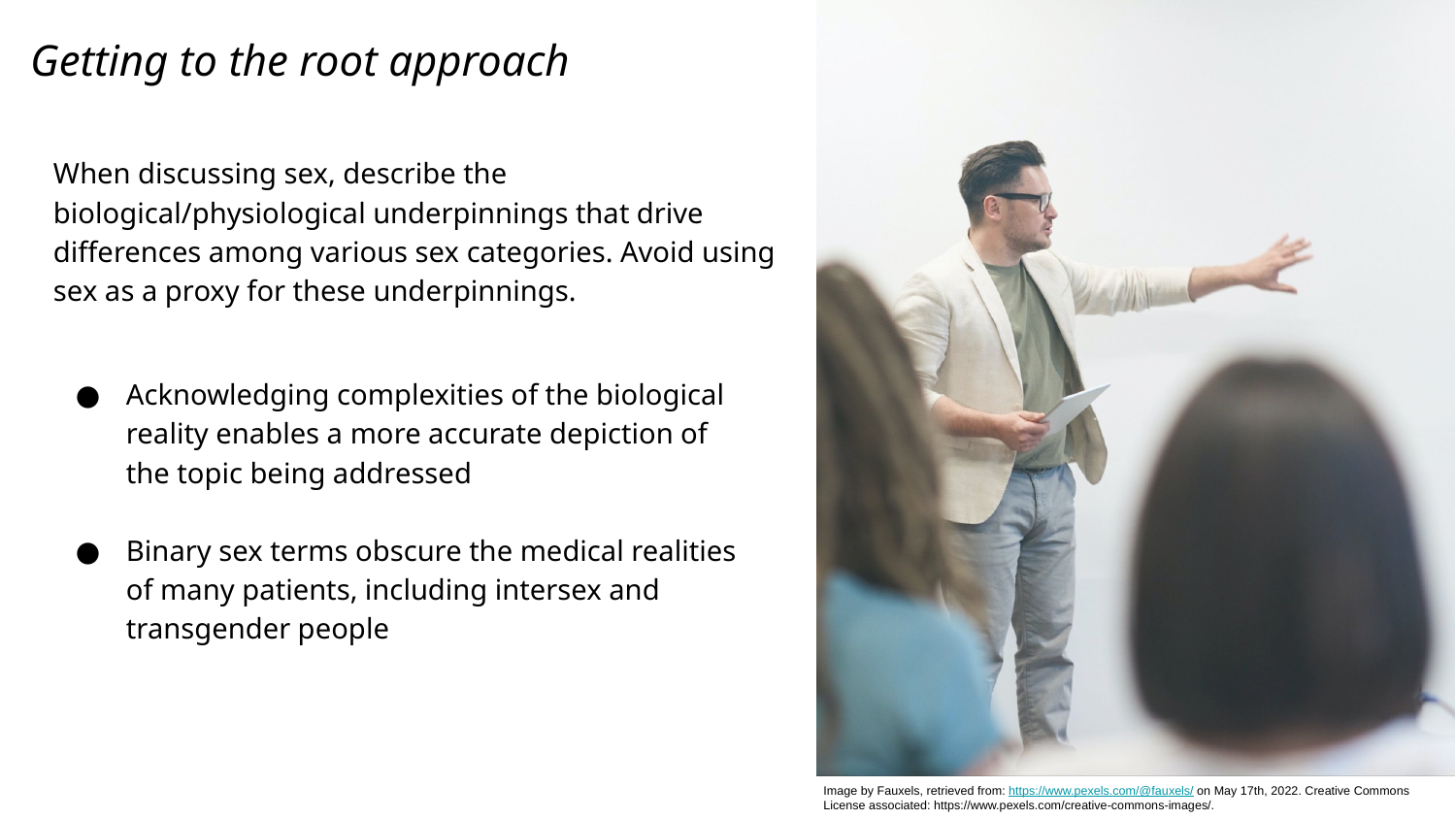

Getting to the root approach
When discussing sex, describe the biological/physiological underpinnings that drive differences among various sex categories. Avoid using sex as a proxy for these underpinnings.
Acknowledging complexities of the biological reality enables a more accurate depiction of the topic being addressed
Binary sex terms obscure the medical realities of many patients, including intersex and transgender people
Image by Fauxels, retrieved from: https://www.pexels.com/@fauxels/ on May 17th, 2022. Creative Commons License associated: https://www.pexels.com/creative-commons-images/.

## Slide 12
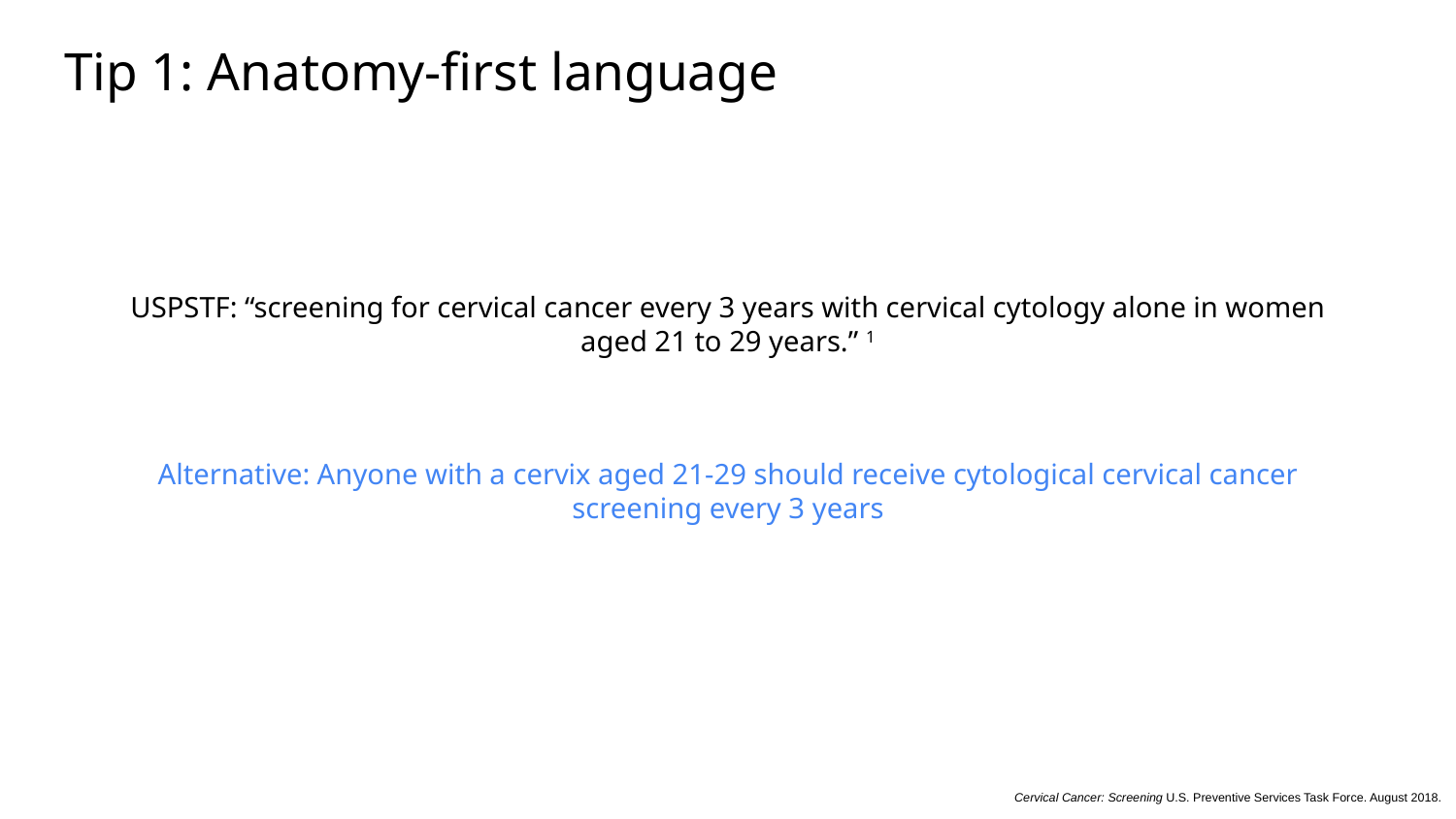

# Tip 1: Anatomy-first language
USPSTF: “screening for cervical cancer every 3 years with cervical cytology alone in women aged 21 to 29 years.” 1
Alternative: Anyone with a cervix aged 21-29 should receive cytological cervical cancer screening every 3 years
Cervical Cancer: Screening U.S. Preventive Services Task Force. August 2018.

## Slide 13
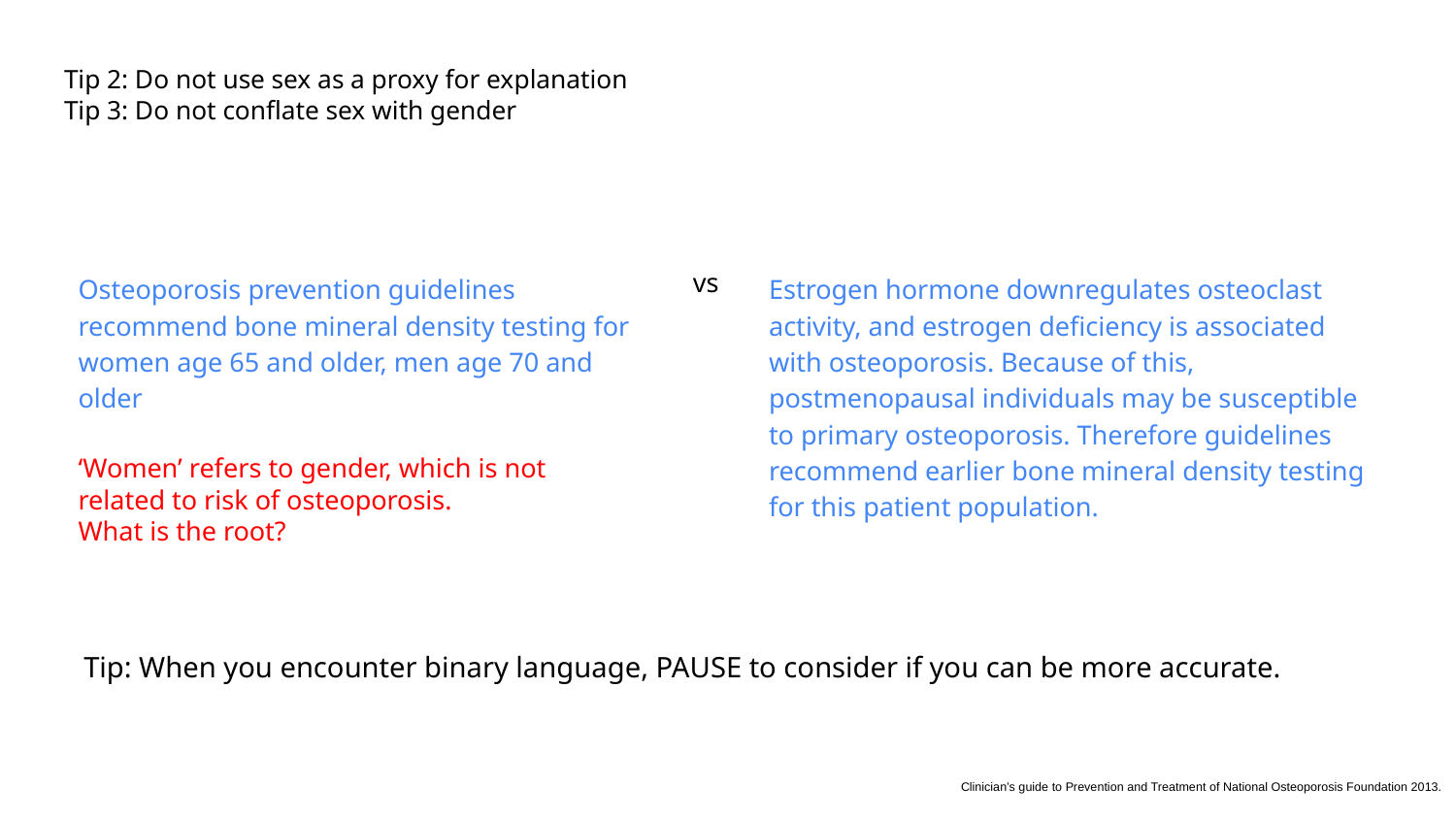

# Tip 2: Do not use sex as a proxy for explanation
Tip 3: Do not conflate sex with gender
vs
Osteoporosis prevention guidelines recommend bone mineral density testing for women age 65 and older, men age 70 and older
Estrogen hormone downregulates osteoclast activity, and estrogen deficiency is associated with osteoporosis. Because of this, postmenopausal individuals may be susceptible to primary osteoporosis. Therefore guidelines recommend earlier bone mineral density testing for this patient population.
‘Women’ refers to gender, which is not related to risk of osteoporosis.
What is the root?
Tip: When you encounter binary language, PAUSE to consider if you can be more accurate.
Clinician’s guide to Prevention and Treatment of National Osteoporosis Foundation 2013.

## Slide 14
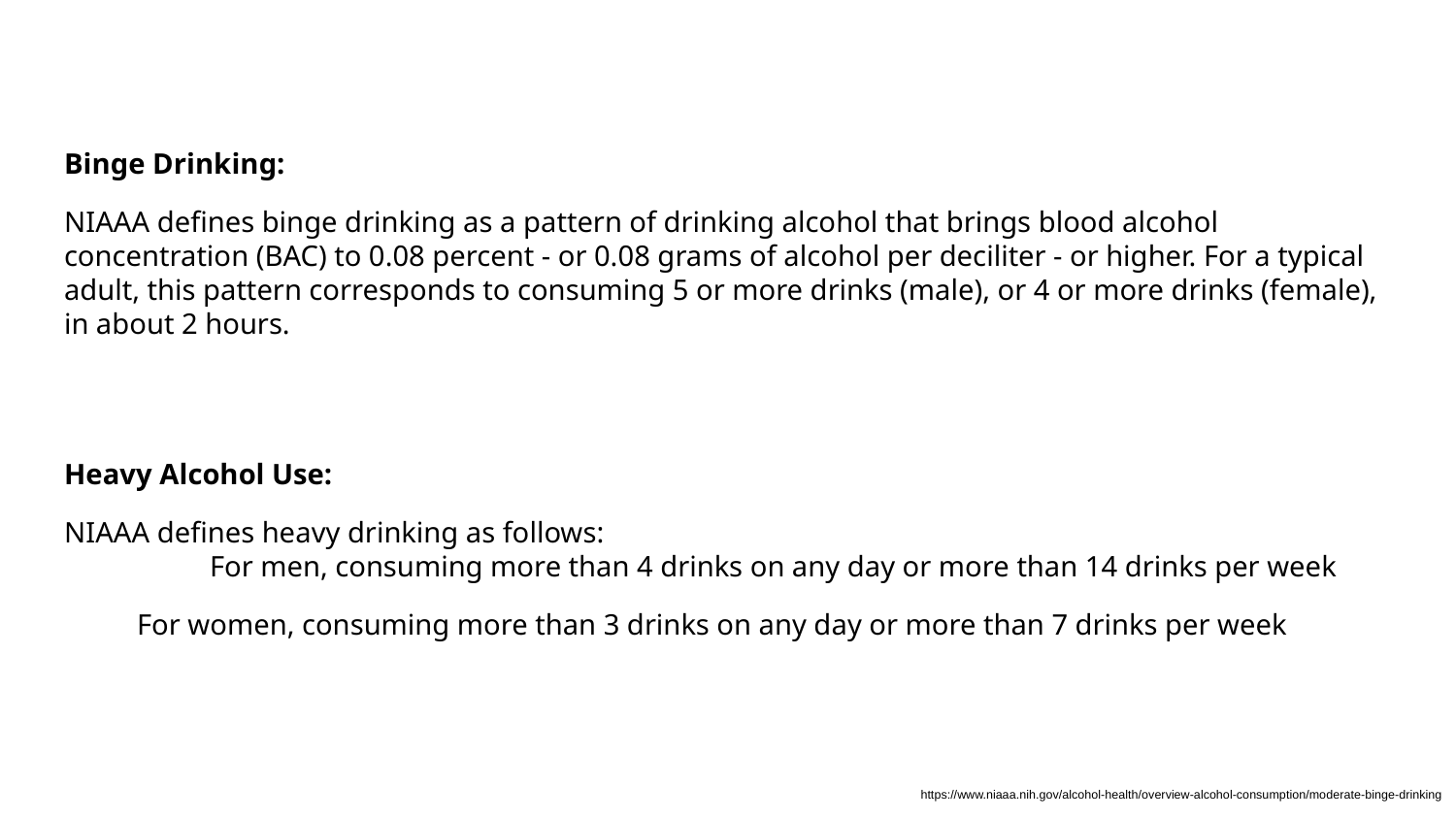

Binge Drinking:
NIAAA defines binge drinking as a pattern of drinking alcohol that brings blood alcohol concentration (BAC) to 0.08 percent - or 0.08 grams of alcohol per deciliter - or higher. For a typical adult, this pattern corresponds to consuming 5 or more drinks (male), or 4 or more drinks (female), in about 2 hours.
Heavy Alcohol Use:
NIAAA defines heavy drinking as follows:
 	For men, consuming more than 4 drinks on any day or more than 14 drinks per week
For women, consuming more than 3 drinks on any day or more than 7 drinks per week
https://www.niaaa.nih.gov/alcohol-health/overview-alcohol-consumption/moderate-binge-drinking

## Slide 15
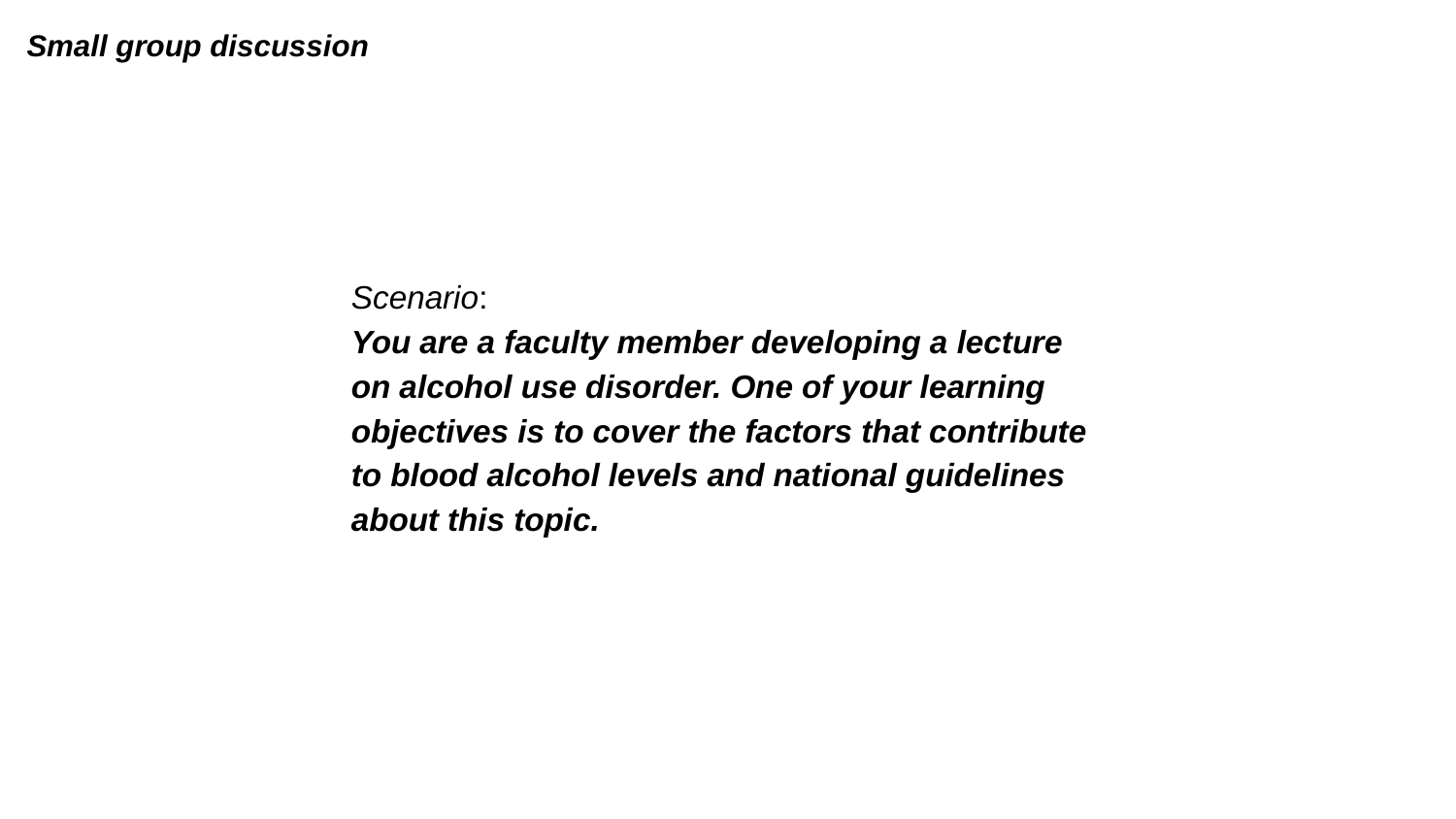

Small group discussion
Scenario:
You are a faculty member developing a lecture on alcohol use disorder. One of your learning objectives is to cover the factors that contribute to blood alcohol levels and national guidelines about this topic.

## Slide 16
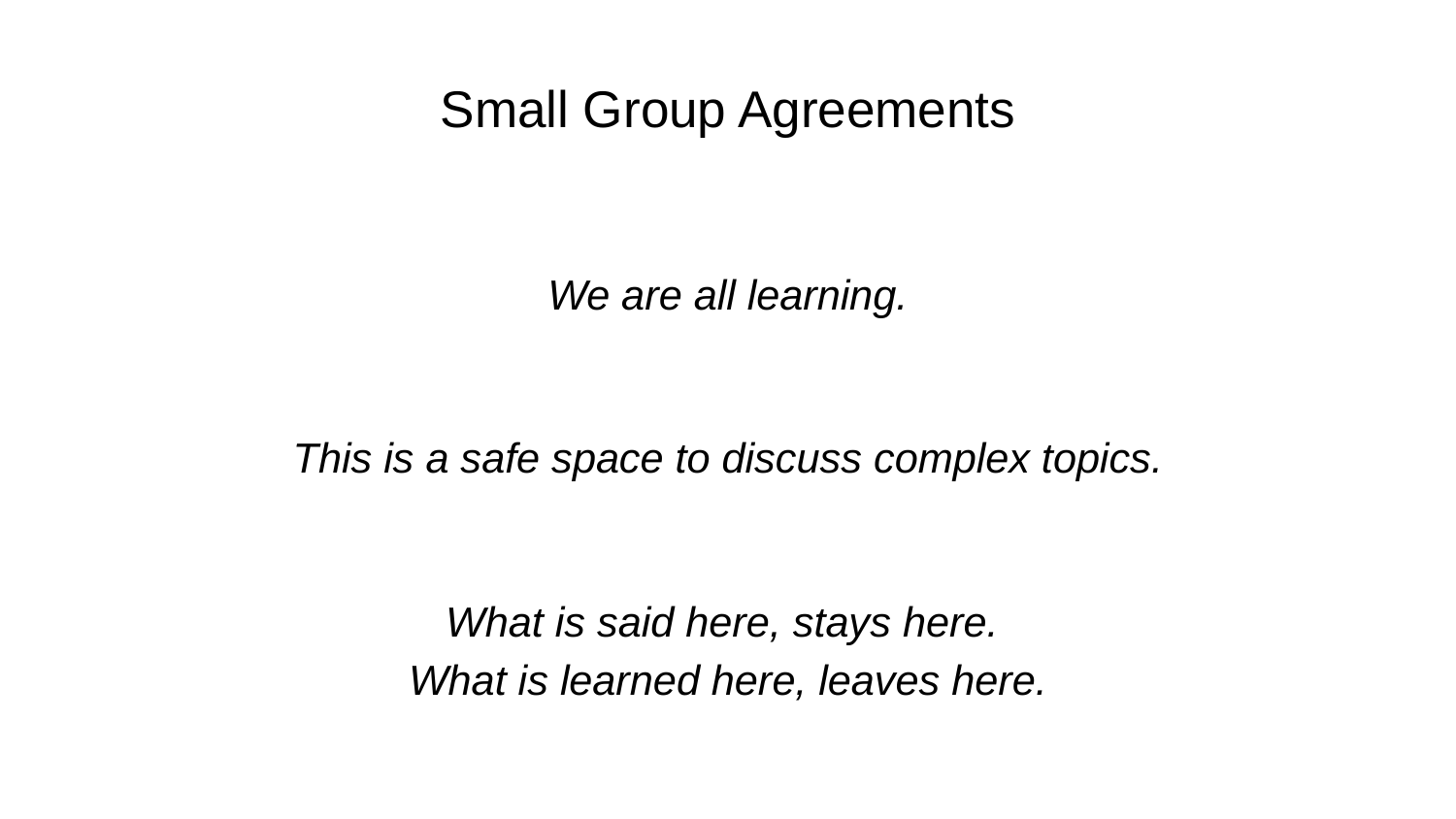

# Small Group Agreements
We are all learning.
This is a safe space to discuss complex topics.
What is said here, stays here.
What is learned here, leaves here.

## Slide 17
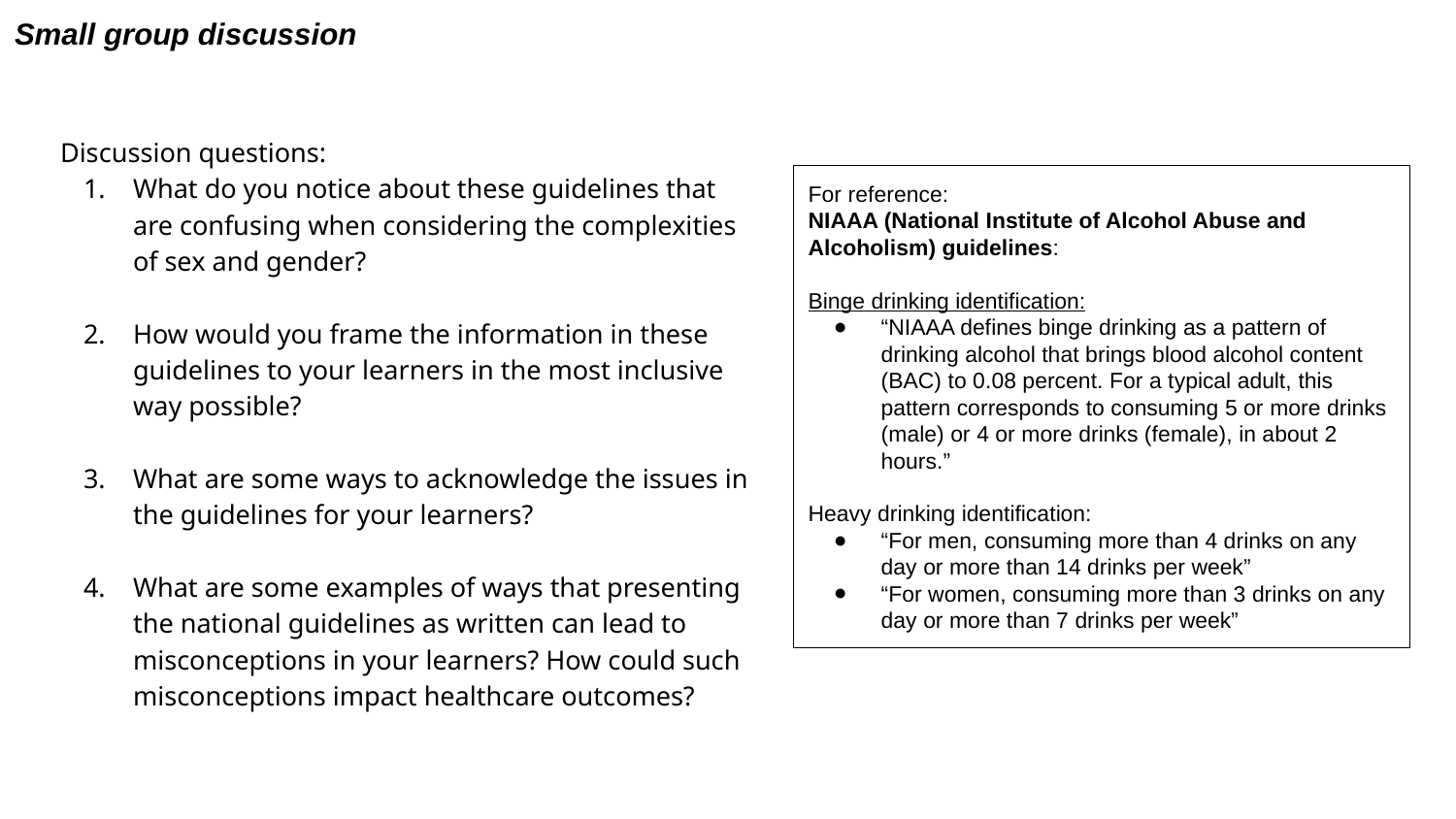

Small group discussion
Discussion questions:
What do you notice about these guidelines that are confusing when considering the complexities of sex and gender?
How would you frame the information in these guidelines to your learners in the most inclusive way possible?
What are some ways to acknowledge the issues in the guidelines for your learners?
What are some examples of ways that presenting the national guidelines as written can lead to misconceptions in your learners? How could such misconceptions impact healthcare outcomes?
For reference:
NIAAA (National Institute of Alcohol Abuse and Alcoholism) guidelines:
Binge drinking identification:
“NIAAA defines binge drinking as a pattern of drinking alcohol that brings blood alcohol content (BAC) to 0.08 percent. For a typical adult, this pattern corresponds to consuming 5 or more drinks (male) or 4 or more drinks (female), in about 2 hours.”
Heavy drinking identification:
“For men, consuming more than 4 drinks on any day or more than 14 drinks per week”
“For women, consuming more than 3 drinks on any day or more than 7 drinks per week”

## Slide 18
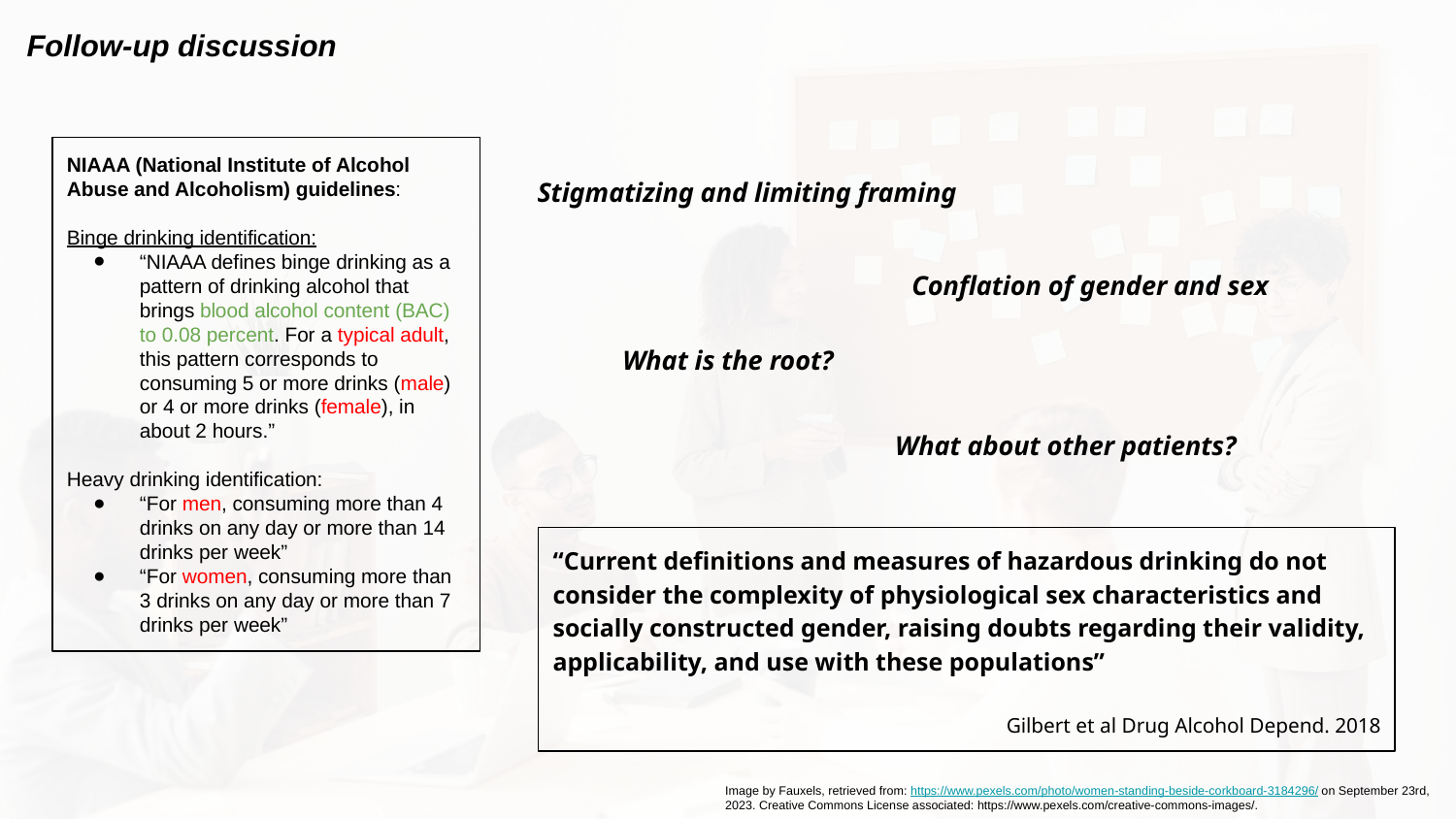

Follow-up discussion
NIAAA (National Institute of Alcohol Abuse and Alcoholism) guidelines:
Binge drinking identification:
“NIAAA defines binge drinking as a pattern of drinking alcohol that brings blood alcohol content (BAC) to 0.08 percent. For a typical adult, this pattern corresponds to consuming 5 or more drinks (male) or 4 or more drinks (female), in about 2 hours.”
Heavy drinking identification:
“For men, consuming more than 4 drinks on any day or more than 14 drinks per week”
“For women, consuming more than 3 drinks on any day or more than 7 drinks per week”
Stigmatizing and limiting framing
Conflation of gender and sex
What is the root?
What about other patients?
“Current definitions and measures of hazardous drinking do not consider the complexity of physiological sex characteristics and socially constructed gender, raising doubts regarding their validity, applicability, and use with these populations”
Gilbert et al Drug Alcohol Depend. 2018
Image by Fauxels, retrieved from: https://www.pexels.com/photo/women-standing-beside-corkboard-3184296/ on September 23rd, 2023. Creative Commons License associated: https://www.pexels.com/creative-commons-images/.

## Slide 19
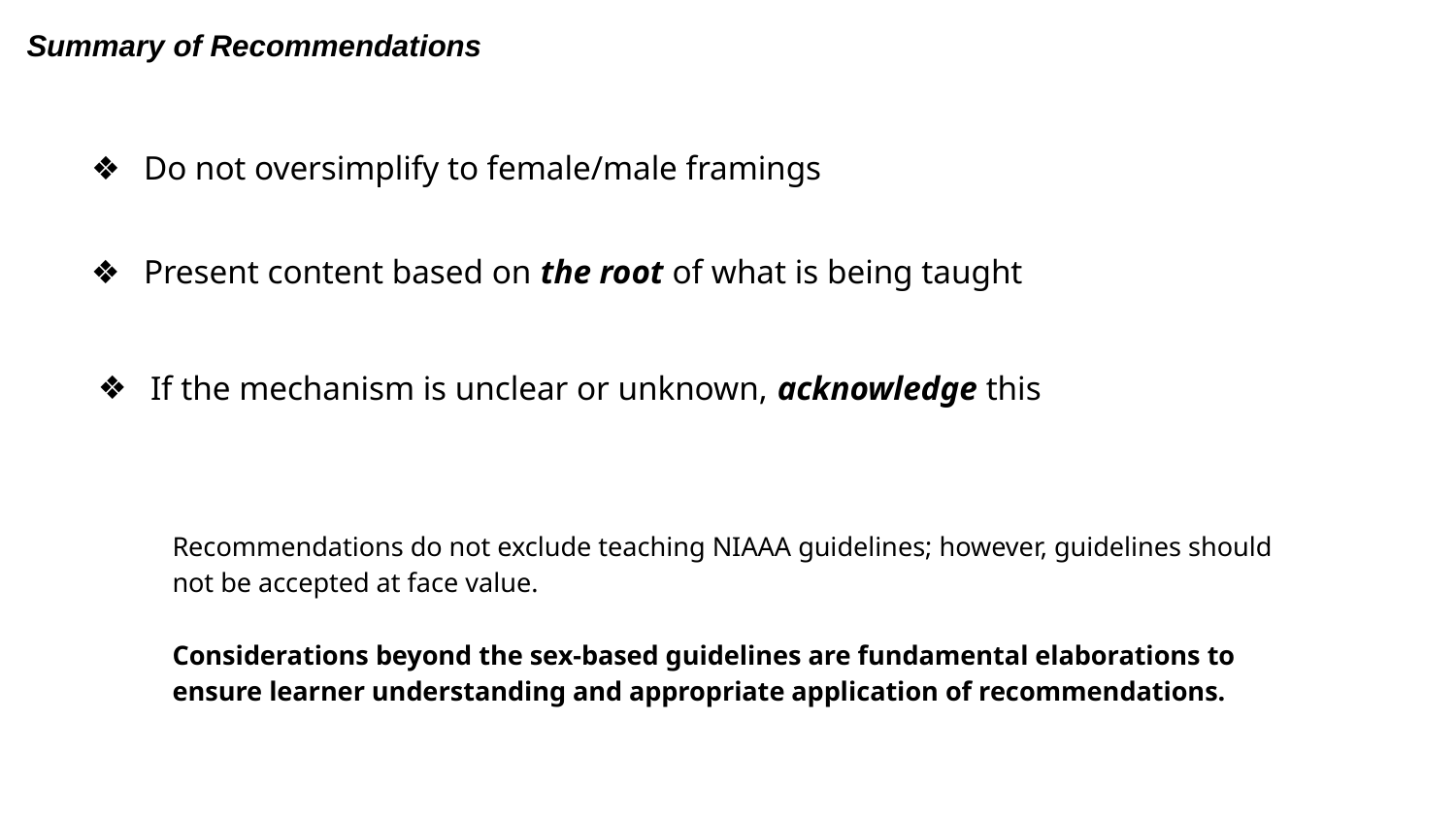

Summary of Recommendations
Do not oversimplify to female/male framings
Present content based on the root of what is being taught
If the mechanism is unclear or unknown, acknowledge this
Recommendations do not exclude teaching NIAAA guidelines; however, guidelines should not be accepted at face value.
Considerations beyond the sex-based guidelines are fundamental elaborations to ensure learner understanding and appropriate application of recommendations.

## Slide 20
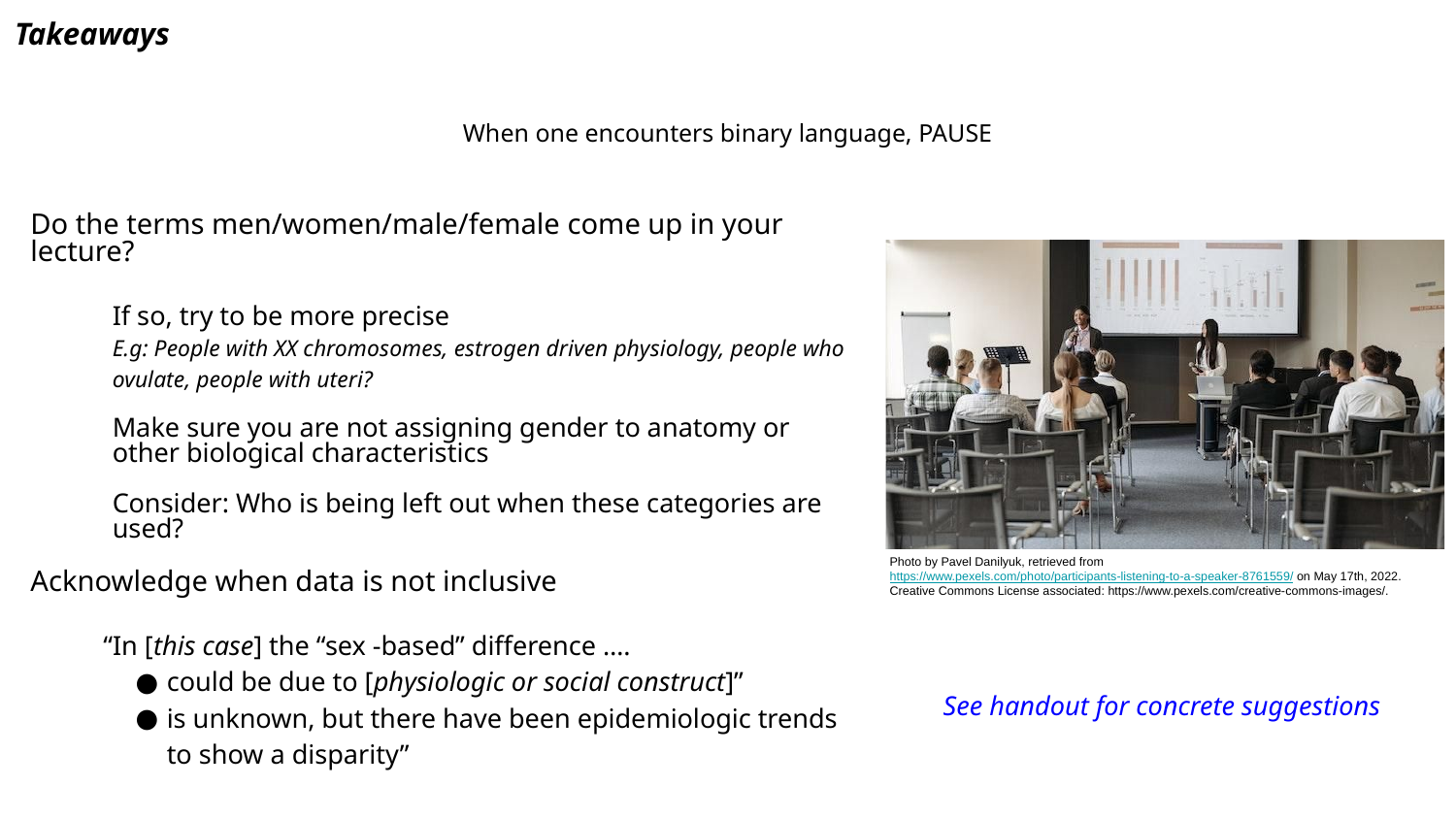

Takeaways
# When one encounters binary language, PAUSE
Do the terms men/women/male/female come up in your lecture?
If so, try to be more precise
E.g: People with XX chromosomes, estrogen driven physiology, people who ovulate, people with uteri?
Make sure you are not assigning gender to anatomy or other biological characteristics
Consider: Who is being left out when these categories are used?
Acknowledge when data is not inclusive
“In [this case] the “sex -based” difference ….
could be due to [physiologic or social construct]”
is unknown, but there have been epidemiologic trends to show a disparity”
Photo by Pavel Danilyuk, retrieved from https://www.pexels.com/photo/participants-listening-to-a-speaker-8761559/ on May 17th, 2022. Creative Commons License associated: https://www.pexels.com/creative-commons-images/.
See handout for concrete suggestions

## Slide 21
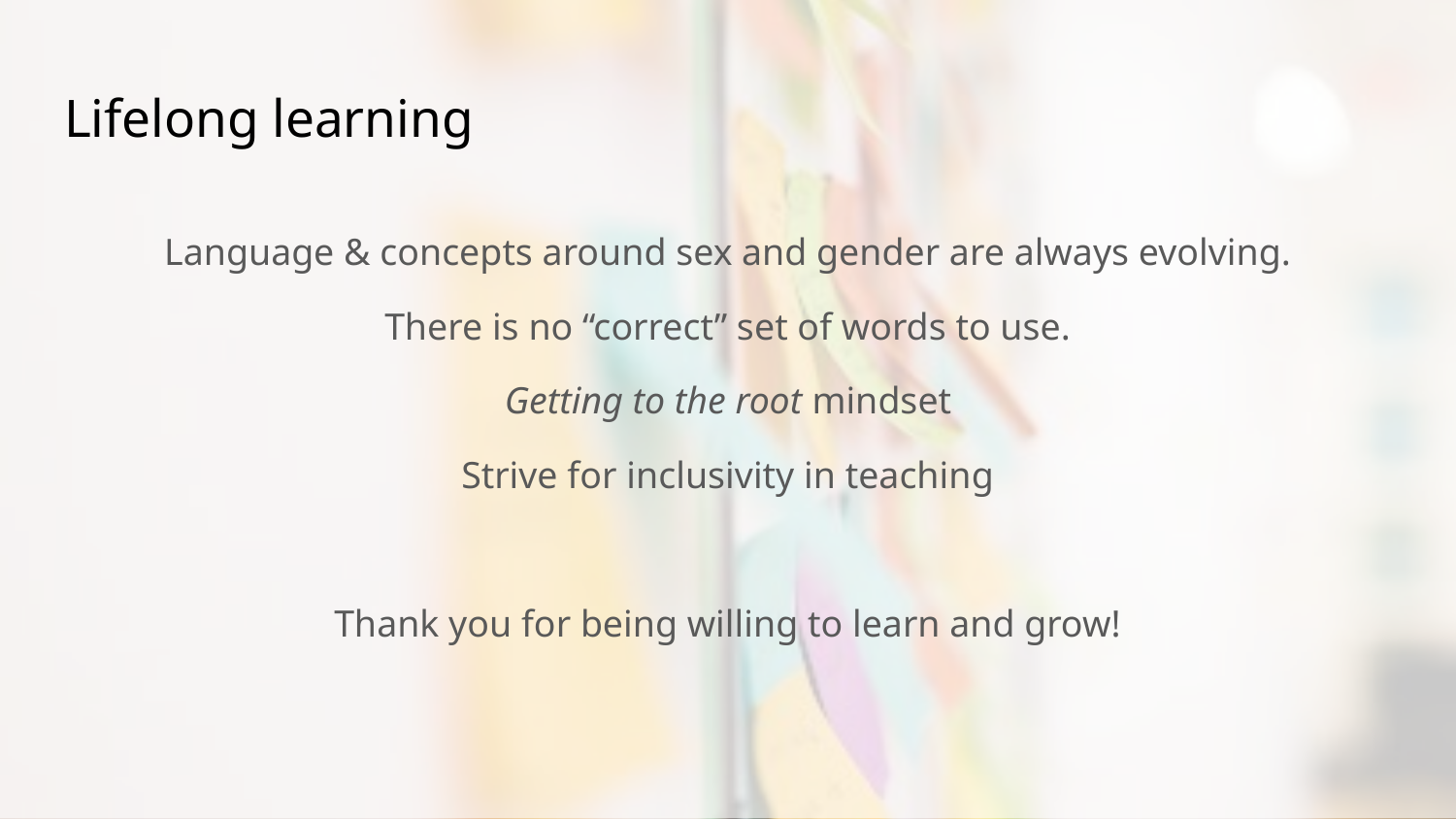

Lifelong learning
Language & concepts around sex and gender are always evolving.
There is no “correct” set of words to use.
Getting to the root mindset
Strive for inclusivity in teaching
Thank you for being willing to learn and grow!

## Slide 22
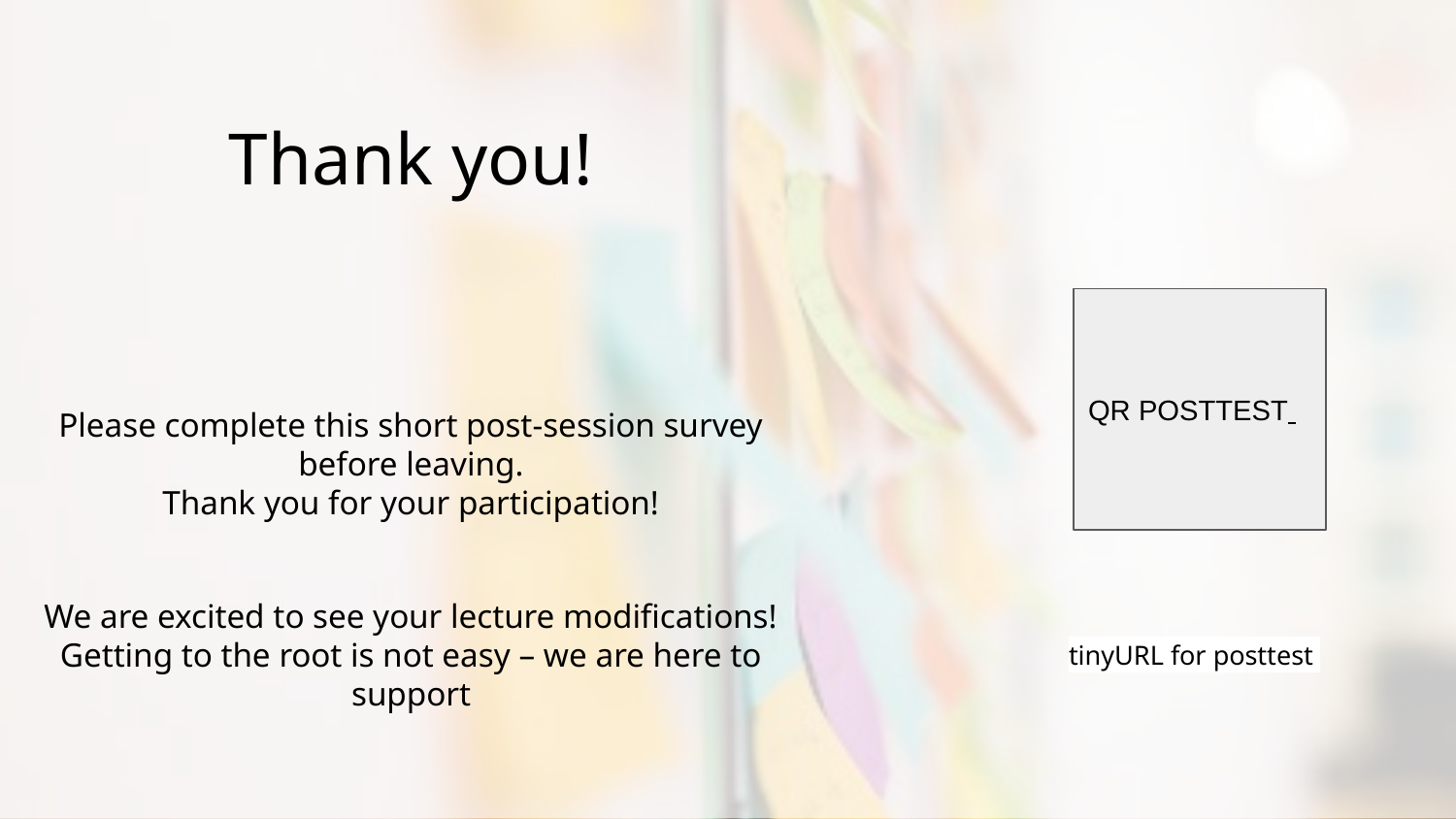

Thank you!
QR POSTTEST
Please complete this short post-session survey before leaving.
Thank you for your participation!
We are excited to see your lecture modifications!
Getting to the root is not easy – we are here to support
tinyURL for posttest
