# Supplementary material for: Faculty Training on Navigating Gender and Sex in Medical Education
Source: MedEdPORTAL. 2024 Aug 13;20:11427. doi: 10.15766/mep_2374-8265.11427 (PMC11319425; doi:10.15766/mep_2374-8265.11427)
Supplement: Supplementary file 1 — Key Terms.docxPresentation With Speaker Notes.pptxSmall-Group Discussion Questions.docxFacilitator Guide.docxHandout Form (Printable Version, Trifold Format).pdfHandout Form (Electronic Version, Standard Format).pdfPre- and Posttraining Survey Forms.docx [file mep_2374-8265.11427-s001.zip › C. Small-Group Discussion Questions.docx]

**Appendix C. Small group discussion questions (approximately 15 minutes, accompanied with a facilitator guide (Appendix D)).** Case presentation and discussion prompts utilizing current NIAAA guidelines on the clinical definitions of binge drinking and heavy alcohol use to help learners consider and apply a getting to the root mindset.

**Lunch and Learn: Gender and Sex Teachings**

**Small Group Discussion**

You are a faculty member developing a lecture on liver physiology. One of your learning objectives is to cover the factors that contribute to blood alcohol levels and national guidelines about this topic. The [National Institute on Alcohol Abuse and Alcoholism (NIAAA) national guidelines](https://www.niaaa.nih.gov/alcohol-health/overview-alcohol-consumption/moderate-binge-drinking) are as follows:

Binge Drinking:

- NIAAA defines binge drinking as a pattern of drinking alcohol that brings blood alcohol concentration (BAC) to 0.08 percent - or 0.08 grams of alcohol per deciliter - or higher. By the NIAAA wording, this corresponds “for a typical adult [as] consuming 5 or more drinks (male), or 4 or more drinks (female), in about 2 hours.”

Heavy Alcohol Use:

- NIAAA defines heavy drinking as follows:
  - For men, consuming more than 4 drinks on any day or more than 14 drinks per week
  - For women, consuming more than 3 drinks on any day or more than 7 drinks per week

**Discussion Questions**

1. What do you notice about these guidelines that are confusing when considering the complexities of sex and gender?
2. How would you frame the information in these guidelines to your learners in the most inclusive way possible?
3. What are some ways to acknowledge the issues in the guidelines for your learners?
4. What are some examples of ways that presenting the national guidelines as written can lead to misconceptions in your learners? How could such misconceptions impact healthcare outcomes?
