# Supplementary material for: Faculty Training on Navigating Gender and Sex in Medical Education
Source: MedEdPORTAL. 2024 Aug 13;20:11427. doi: 10.15766/mep_2374-8265.11427 (PMC11319425; doi:10.15766/mep_2374-8265.11427)
Supplement: Supplementary file 1 — Key Terms.docxPresentation With Speaker Notes.pptxSmall-Group Discussion Questions.docxFacilitator Guide.docxHandout Form (Printable Version, Trifold Format).pdfHandout Form (Electronic Version, Standard Format).pdfPre- and Posttraining Survey Forms.docx [file mep_2374-8265.11427-s001.zip › D. Facilitator Guide.docx]

**Appendix D. Facilitator guide (approximately 15 minutes, accompanied with a small group discussion questions (Appendix C)).** A facilitator guide with a suggested detailed timeline of the hour-long training session, as well as suggested comments (blue text) to help guide the small group case presentation discussion (black text). Further suggested responses to potential questions during the small group discussion are also included below (italicized black and blue text).

**Lunch and Learn**

**Timeline:**

12:00-12:05: Introduction, pre-training survey, learning objectives

12:05-12:20: Context of LGBTQIA+ healthcare history in the United States

12:20-12:30: Gender and sex in medical education, getting to the root mindset of gender and sex

12:30-12:35: Case presentation

12:35-12:50: Facilitated small groups

12:50-12:55: Discussion wrap-up

12:55-1:00: Take home messages, closing, post-training survey

**Divide into small groups of 5-8 participants**

- **Groups can be either in person or on zoom. Hybrid within the same group would be challenging**
- **Facilitators: At least one faculty member, one student per group**

#### **Scenario 1:**

(Facilitators Guide information is in blue. Participants receive the same document without the blue text.)

For facilitators: The goal of this scenario is to explore the situation in which a lecturer encounters confusing or misleading sex/gender information in the context of expert resources. What is written in blue are some possible directions the discussion should follow. If these points do not naturally come up in the conversation, we recommend you read it to the group so that everyone learns the same points.

You are a faculty member developing a lecture on liver physiology. One of your learning objectives is to cover the factors that contribute to blood alcohol levels and national guidelines about this topic. The [National Institute on Alcohol Abuse and Alcoholism (NIAAA) national guidelines](https://www.niaaa.nih.gov/alcohol-health/overview-alcohol-consumption/moderate-binge-drinking) are as follows:

Binge Drinking:

- NIAAA defines binge drinking as a pattern of drinking alcohol that brings blood alcohol concentration (BAC) to 0.08 percent - or 0.08 grams of alcohol per deciliter - or higher. By the NIAAA wording, this corresponds “for a typical adult [as] consuming 5 or more drinks (male), or 4 or more drinks (female), in about 2 hours.”

Heavy Alcohol Use:

- NIAAA defines heavy drinking as follows:
  - For men, consuming more than 4 drinks on any day or more than 14 drinks per week
  - For women, consuming more than 3 drinks on any day or more than 7 drinks per week

**Small group discussion:**

1. What do you notice about these guidelines that are confusing when considering the complexities of sex and gender?
   - Conflation of sex and gender within the same guidelines
   - Unclear how the guidelines apply for intersex or transgender people
   - Stigmatizing framing of viewing certain adults as ‘typical’ and others ‘atypical’ (consider body diversity)
2. How would you frame the information in these guidelines to your learners in the most inclusive way possible?
   - The educator should explain the scientific underpinnings of the guidelines (i.e. what drives the metabolic processing of alcohol that differs in “females” and “males”?)
   - NIAAA guidelines categorize patients by ‘male and female’ as a proxy of **volume of distribution**, which seems to be the most established bodily influence on blood alcohol content (BAC) levels
     - Alcohol is hydrophilic, and does not absorb into fat. Therefore people with greater volume of distribution (higher water:fat ratio) have more volume into which alcohol can absorb and experience less extreme changes in blood alcohol content (BAC) with the same alcohol consumption, and thereby have a lower risk of experiencing toxicity from alcohol metabolites.^3^
       - National guidelines allow more drinks for bodies with higher volumes of distribution
     - Examples of patients that fall outside the recommendations:
       - **Estrogen hormone replacement therapy as part of gender affirming care:** Exogenous estrogen use in people did not complete estrogen-driven puberty undergo a redistribution of their body composition, notably with a loss of lean mass and increase in fat distribution^1^ -> patients experiencing changes in body proportions due to exogenous estrogen use have an increased sensitivity to alcohol, and theoretically should have lower alcohol drinking guidelines
       - **Body habitus with an increased proportion of fat:** Increase in fat relative to body size is associated with reduced BAC changes with alcohol consumption^2^ -> patients with an increased proportion of fat theoretically have a decreased sensitivity to alcohol, and theoretically should have higher alcohol drinking guidelines
     - There are other influences on BAC, albeit some are less established and they seem to go beyond typical lecture teaching points
       - **Gastric alcohol dehydrogenase (ADH) activity** influences how much alcohol reaches the intestinal tract and is absorbed into the bloodstream, and literature has studied differences in gastric ADH activity related to sex assigned at birth, hormone profile, age, chronicity of alcohol intake^4,5,6^ -> greater gastric ADH activity leads to lower BAC levels after oral alcohol intake
       - Ethanol excretion rates are similar among all people, suggesting that those with less volume of distribution (“females”) have increased hepatic metabolism activity^5^

**Questions to pose**: What does this tell us about advice about alcohol consumption for individuals that fall outside the male/female binary, or for patients on hormone replacement therapy? What about individuals whose body physiology falls outside the range assumed by the guidelines? (e.g. male individuals with high body fat percentage; people with extremely low body fat)

1. What are some ways to acknowledge the issues in the guidelines for your learners?
   - Explicitly point out the conflation of sex and gender
   - Discuss that “expert” information is often incomplete either due to how the data was studied (limited study populations) or because it is common to use male/female as a proxy for other physiologic factors.
2. What are some examples of ways that presenting the national guidelines as written can lead to misconceptions in your learners? How could such misconceptions impact healthcare outcomes?
   - By simplifying alcohol use guidelines to male/female, learners will not appreciate the factors that underpin the guidelines (e.g., metabolism of alcohol and how it differs among individuals, volume of distribution, liver mass and enzyme activity relative to volume of distribution).
   - Guidelines divided by male/female also leads to confusion about what advice to give to people who fall outside the binary male/female or “typical” body composition of those categories.

**This section is not included in the group handout. Here we list some potential comments/inquiries from the small group and our recommended responses:**

*“These are the federal guidelines, they’re the best we have and what we should teach.”*

- - *These may be the federal guidelines for now, but we also recognize the many ways in which they are not sufficient. As physicians we are always pushing ourselves to learn more and do better.*

*“We just don’t have the time to acknowledge all of the nuances of sex in every lecture, it’s impractical.”*

- - *If you know in your teaching these types of examples will come up frequently, consider starting the course/lecture with a more in-depth statement about how sex is not a binary and acknowledge the physiological and sociocultural underpinnings of differences between “sexes” and refer back to this statement when relevant throughout the course. Throughout the course, continue to try to “get to the root” to describe why a given difference is noted by sex or gender.*

*“What terminology can I use when presenting the guidelines to not offend anyone?”*

- - *When presenting guidelines, one might say sex is not a simple binary. Although the data were collected as if sex is a binary, we do not know the identities or all nuances of the physiologies of the people who participated in the research. Follow that statement with: “for our purposes today, we are going to discuss these data as published.”*

*“What if the underlying physiological causes are unknown, what am I supposed to say?”*

- - *It is not uncommon for us not to fully understand the root causes of many differences noted between sexes.*
  - *The most important thing is to acknowledge that sex is not a binary. Although the primary data are typically collected as these categories are clearly separate, we do not know the identities or all nuances of the physiologies of the people who participated in the research. Then: “we do not yet know the underlying physiological mechanisms that drive these observed differences between parts of the population.”*

**References**

1. Spanos C, Bretherton I, Zajac JD, Cheung AS. Effects of gender-affirming hormone therapy on insulin resistance and body composition in transgender individuals: A systematic review. World J Diabetes. 2020;11(3):66-77. doi:10.4239/wjd.v11.i3.66
2. Wingren CJ, Ottosson A. The association between obesity and lethal blood alcohol concentrations: a nationwide register-based study of medicolegal autopsy cases in Sweden. Forensic Sci Int. 2014;244:285-288. doi:10.1016/j.forsciint.2014.09.012
3. Mumenthaler MS, Taylor JL, O'Hara R, Yesavage JA. Gender differences in moderate drinking effects. Alcohol Res Health. 1999;23(1):55-64.
4. Baraona E, Abittan CS, Dohmen K, et al. Gender differences in pharmacokinetics of alcohol. Alcohol Clin Exp Res. 2001;25(4):502-507
5. Parlesak A, Billinger MH, Bode C, Bode JC. Gastric alcohol dehydrogenase activity in man: influence of gender, age, alcohol consumption and smoking in a caucasian population. Alcohol Alcohol. 2002;37(4):388-393. doi:10.1093/alcalc/37.4.388
6. Thomasson HR. Gender differences in alcohol metabolism. Physiological responses to ethanol. Recent Dev Alcohol. 1995;12:163-179. doi:10.1007/0-306-47138-8_9
