# Supplementary material for: Faculty Training on Navigating Gender and Sex in Medical Education
Source: MedEdPORTAL. 2024 Aug 13;20:11427. doi: 10.15766/mep_2374-8265.11427 (PMC11319425; doi:10.15766/mep_2374-8265.11427)
Supplement: Supplementary file 1 — Key Terms.docxPresentation With Speaker Notes.pptxSmall-Group Discussion Questions.docxFacilitator Guide.docxHandout Form (Printable Version, Trifold Format).pdfHandout Form (Electronic Version, Standard Format).pdfPre- and Posttraining Survey Forms.docx [file mep_2374-8265.11427-s001.zip › _Educational Summary Report_11427.pdf]

MedEdPORTAL® The AAMC Journal of Teaching and Learning Resources

Original Publication Open Access

Faculty Training on Navigating Gender and Sex in Medical Education

Benjamin Crosby*†, Hannah Dumas, MPH†, Janet Monroe, Fredric Fabiano, MS, Isabelle Gell-Levey, Christopher Noyes, Kikuye Sugiyama, Jennifer Siegel, MD, Angelique Harris, PhD, Carl Streed Jr, MD, MPH, Ann C. Zumwalt, PhD

*Corresponding author: bencrosby22@gmail.com

†Co-primary author

Abstract

Introduction: Language that assumes gender and sex are binary and aligned is pervasive in medicine and is often used when teaching on physiology and pathology. Information presented through this lens oversimplifies disease mechanisms and poorly addresses the health of gender and sexually diverse (GSD) individuals. We developed a training session to help faculty reference gender and sex in a manner that would be accurate and inclusive of GSD health. **Methods:** The 1-hour session for undergraduate and graduate medical educators highlighted cisgender and binary biases in medical teachings and introduced a getting-to-the-root mindset that prioritized teaching the processes underlying differences in disease profiles among gender and sex subpopulations. The training consisted of 30 minutes of didactic teaching and 20 minutes of small-group discussion. Medical education faculty attended and self-reported knowledge and awareness before and after the training. Results were compared using paired *t* tests. Expenses included fees for consultation and catering. **Results:** Forty faculty participated (pretraining survey $n = 36$, posttraining survey $n = 21$). After the training, there was a significant increase in self-reported awareness of the difference between gender and sex ($p = .002$), perceived relevance of gender to teachings ($p = .04$), and readiness to discuss physiological drivers of sex-linked disease ($p = .005$). **Discussion:** Participants reported increased understanding and consideration of gender and sex in medical education; feedback emphasized a desire for continued guidance. This easily adaptable session can provide an introduction to a series of medical teachings on gender and sex.

Keywords

Gender and Sexual Diversity, Sexual and Gender Minorities, Clinical Reasoning/Diagnostic Reasoning, Cultural Competence, Differences of Sexual Development, Faculty Development, Gender Identity, Health Equity, LGBTQ+, Diversity, Equity, Inclusion

Educational Objectives

By the end of this session, faculty learners will be able to:

1. Describe the pervasiveness of cisgender and gender binary biases in medical education.
2. Appreciate how use of the terms *women/female* and *men/male* in medical training can exclude or hold ambiguous applicability for transgender and gender-diverse people, thereby warranting clarification and context.
3. Begin to describe and teach the pathophysiological factors that drive differences in disease profiles among populations that have previously been grouped into gender and sex categories in an undergraduate/graduate medical education setting.

Citation:
Crosby B, Dumas H, Monroe J, et al. Faculty training on navigating gender and sex in medical education. *MedEdPORTAL*. 2024;20:11427. https://doi.org/10.15766/mep_2374-8265.11427

Introduction

Binary gender and sex categories are utilized throughout medical education to teach characteristics, processes, and disease patterns. The practice of teaching disease prevalence as linked to gender and sex (e.g., "Disease X is typically found in women/females ages 30-50") implies that gender/sex is the risk factor for the disease, rather than more explicitly describing the gender-/sex-linked mechanisms that drive the disease patterns. This practice obscures the mechanisms that drive disease patterns and ultimately limits learners' ability to interpret variations observed clinically (Table 1).

Furthermore, many clinical guidelines and epidemiological patterns are framed in a manner that assumes all individuals' sex assigned at birth and gender identity are congruent

Copyright © 2024 Crosby et al. This is an open-access publication distributed under the terms of the Creative Commons Attribution license.

Table 1. Getting to the Root of Gender and Sex Examples

Typical Framing	Implicit Issues	More Inclusive or Descriptive Framing Example
Disease prevalence: Systemic lupus erythematosus is nine times more prevalent in females.	Absence of further explanation regarding underlying disease mechanism limits learners' ability to conceptualize whether increased prevalence is relevant to people who identify as women, who are AFAB, who may or may not be receiving any form of hormone therapy or gender-affirming surgery.	Systemic lupus erythematosus is nine times more prevalent in females. This disparity is thought to be partly hormonally mediated by estradiol, though its mechanism remains enigmatic as literature has also noted contribution via X chromosome and sex-related cytokine pathways.
Clinical guidelines: Heavy drinking is defined as consuming more than four drinks on any day or more than 14 drinks per week for men, or more than three drinks on any day or more than seven drinks per week for women.	Does not indicate whether rationale is related to sex-linked biological drivers, body size, gendered lifestyle/behavioral/other social factors, or something else.<br>Obscures potential lessons on the physiological drivers of blood alcohol content and prevents learners from developing a mental schema to understand individual susceptibility.<br>Provides no guidance on how to apply the guidelines to transgender, nonbinary, other gender-diverse, or intersex patients.	Teach the national guidelines as provided, with the added nuances: "Men" is not the same as "assigned male at birth." The reason for this disparity is primarily related to differences in body size and fat:water ratio, whereby people with smaller body size and greater fat:water content are more susceptible to changes in blood alcohol content.
Clinical guidelines: Women ages 21-29 should be screened for cervical cancer with cervical cytology alone every 3 years.	Does not address how to apply guidelines for:<br>• People with a cervix who do not identify as women (e.g., transgender men, nonbinary AFAB people).<br>• People who identify as women who do not have a cervix (postsurgical, differences in sex development).	People with a cervix ages 21-29 should be screened for cervical cancer with cervical cytology alone every 3 years.

Abbreviation: AFAB, assigned female at birth.

(cisgender) and strictly male or female (binary). These practices are problematic because these framings exclude gender-diverse people, such as those whose gender identity does not align with their sex assigned at birth, and people with differences of sex development or intersex characteristics (see Appendix A for definitions). Such framings essentially negate the existence of gender-diverse and intersex patients and inadequately prepare trainees to provide appropriate care for these patients. This concern is particularly poignant in light of extensive evidence that provider-driven stigma against and uninformed care for gender and sexually diverse (GSD) people is one of the the greatest barriers to care for GSD patients.[1-4]

Getting to the Root of Gender and Sex

In recognition of the problems of cisgender bias described above, we developed a training session to guide faculty on how to more inclusively frame information related to gender or sex. The goals of this training are to demonstrate the pervasiveness of binary framings of gender and sex in medical education, explain why such framings limit learners' understanding and exclude GSD people, and suggest alternative framings using typical teaching examples. Grounding medical teachings in lessons that explain the biological drivers (e.g., estrogen/testosterone levels, presence/absence of certain organs, chromosome expression, behavioral or environmental factors, etc.) enables educators to teach the mechanisms that underlie observed differences rather than using sex or gender categories as proxies for these mechanisms. This training is designed to help faculty reframe lessons to prioritize the mechanisms (the root) that drive differences between various gender and sex patient populations, rather than simply ascribing differences to binary gender/sex demographic categories. The dissection and intentional reframing of traditional cisnormative and binary teachings to physiology-rooted models serve the understanding of all patient health and, in particular, GSD populations.

Methods

This investigation was deemed exempt by the Boston University Medical Campus Institutional Review Board (H-42554).

Overview

This 1-hour session was intended for faculty teaching in any part of an undergraduate or graduate medical curriculum. It included a brief introduction to the history of GSD health care in the United States, an overview of the pervasiveness of cisnormative and binary categorizations of gender and sex in medical education, a dialogue on how the assumptions underlying these framings negate the existence of GSD identities, and a case presentation with facilitated small-group discussions. At our institution, the session was developed and presented by both faculty and students to demonstrate how both groups could benefit from this approach. We advertised this voluntary and stand-alone session on medical faculty email distribution lists. Expenses included lunch catering and a consultation fee, which may not

Copyright © 2024 Crosby et al. This is an open-access publication distributed under the terms of the Creative Commons Attribution license.

be an additional expense for other groups that consider adapting this training without further consultation input. No incentive was provided for completion of pre- or postsurvey forms.

The training was designed to have the option to be delivered in person, remotely, or in a hybrid format, and facilitators could mediate either live or remote audiences. When delivered at our institution, participants attended both remotely and in person.

Prework: vocabulary: To save time during the workshop, we asked that participants review a video defining core terminology before the workshop.[5] Specifically, this prework defined and described the differences between gender and sex; provided brief definitions of gender identity and pronoun usage, differences of sex development/intersex features, and sexual orientation; and offered examples across the spectrums of each category.

Part 1: didactic (Appendix B): The didactic portion of the session was approximately 30 minutes long and consisted of three parts: (1) contextualization of the training in the current state of GSD health/health care in the United States, (2) illumination of problematic issues with the typical framings of sex and gender, and (3) description of a getting-to-the-root mindset.

At our institution, the training was presented by a consultant, faculty member, and medical student, although the training could be implemented by any combination of roles. The consultant was an expert on GSD health issues who also provided insights during the development of the session. In our training, the consultant introduced the session with a presentation on its relevance in the context of the history of GSD health care in the United States, with a focus on the evolution of the medical community's understanding of GSD health and evidence connecting GSD disparities with inadequate care within the medical system. These lessons were used to frame why it was incumbent upon current medical educators to improve the inclusivity of medical training. While this content was an important part of the training, it was not essential that it be presented by an external consultant or expert on GSD health.

A faculty member (author Ann C. Zumwalt) then reviewed fundamental vocabulary related to gender and sex and highlighted how these concepts appeared in medical education. A primary objective of this section was to demonstrate that not only were gender and sex ubiquitous in medical education but that they were typically presented without questioning the underlying assumptions of cisnormative or binary perspectives. This section also highlighted how gender and sex were frequently conflated and delineated the repercussions of these practices.

In the final component of the didactic section, a medical student (author Benjamin Crosby) introduced a getting-to-the-root approach to gender and sex, defining this approach and describing techniques to apply it, including avoiding using sex as a proxy, when and how to prioritize anatomy-first language, and avoiding conflations of gender and sex. Practical tips for educators to monitor and modify their practices during teaching were presented throughout this portion of the workshop.

Part 2: small-group session (Appendices C and D): The workshop participants were then divided into small groups and worked through a 15-minute learning case. This portion of the training was designed for groups of six to eight people with two to three facilitators per group. At our institution, we formed three discussion groups, two in person and one remote via teleconference, each with approximately eight participants and facilitated by one faculty and two student team members.

Participants were provided with current national diagnosis guidelines for binge drinking and heavy alcohol use[6] and a series of discussion prompts designed to foster a critical examination of the biases inherent to how gender and sex were referred to in the guidelines, groups considered, and ways to modify presentation of this information to be more inclusive (Appendix C).

Facilitators received a facilitator guide (Appendix D) detailing the key points to cover for each of the discussion questions to guide group conversation and ensure learning objectives were met in all groups. The facilitator guide also included hypothetical comments or questions that might arise, such as "What terminology can I use when presenting guidelines to not offend anyone?" and "I don't understand how I can build this into my teaching," along with suggested responses.

Part 3: large-group discussion: The small groups reconvened after the breakout sessions to discuss and review what participants had learned. The workshop concluded with a summary of recommendations for modifying teaching practices to apply a getting-to-the-root approach to gender and sex, including techniques to address topics where the mechanism driving differences between gender and sex populations was unknown. Participants were provided with a handout outlining these recommendations and summarizing key takeaways from the session (Appendices E and F).

Copyright © 2024 Crosby et al. This is an open-access publication distributed under the terms of the Creative Commons Attribution license.

Analysis
Before and after the training, participants were surveyed on self-reported knowledge of the difference between gender and sex, awareness of GSD health inequities, perceived relevance of sex and gender to course teachings, and comfort discussing factors underlying differences in disease incidence among different gender and sex groups (Appendix G). Respondents also indicated their current teaching responsibilities, prior experiences with GSD training, and optional demographic information regarding gender, race and ethnicity, and lesbian, gay, bisexual, transgender, queer/questioning (LGBTQ+) identity. Participants completed the online surveys using links provided before and at the closing of the session (Qualtrics XM). Surveys were labeled with a unique and anonymous participant-chosen identifier to allow matching of pre- and posttraining responses.

Survey responses were analyzed using Excel and SPSS 27 (IBM). Descriptive survey answers were scored on a 5-point Likert scale (1 = *not at all,* 2 = *slightly,* 3 = *moderately,* 4 = *very,* 5 = *extremely*) for analysis. We assessed distribution and variance patterns using normal Q-Q plots and *F* tests, which demonstrated normal distribution and equal variances in the datasets. Subsequent comparisons of pre- and postsurvey data were made using paired *t* tests with equal variance ($\alpha = .05$, two-tailed) and paired power analyses (single power value = .80, two-tailed; Pearson product-moment correlation coefficient = .5).

Results

The training session was attended by 40 faculty members. The pretraining survey was completed by 36 faculty (90% response rate), of whom 12 (33%) taught only in the preclerkship curriculum, four (11%) taught only in the clerkship curriculum, and 15 (42%) taught in both curricula. Five participants (14%) reported that they did not teach in either curriculum. The majority of respondents did not identify as LGBTQ+ (83%), and no respondent identified as nonbinary, though with the caveat that this was an optional demographic question that may have been skipped to avoid compromising anonymity. The majority of respondents had previous training (formal and/or informal) on GSD-related topics (89%; Table 2).

Table 2. Faculty Training Audience Demographic Information ($N = 36$)

Demographic	No. (%)
Teaching context	
Clinical	4 (11)
Clinical and preclerkship	15 (42)
Preclerkship	12 (33)
Neither	5 (14)
Previous gender and sexually diverse training	
Formal	17 (40)
Informal	21 (49)
None	5 (12)
LGBTQ+ identity	
Yes	6 (17)
No	29 (83)

Prior to the training, most faculty self-reported that they felt moderately or very knowledgeable about the difference between gender and sex (48% and 33%, respectively; Table 3). While most faculty felt topics of sex were moderately, very, or extremely relevant to their teachings (43%, 24%, and 19%, respectively), faculty were more divided about the relevance of gender, with respondents indicating slight (29%), moderate (19%), very (33%), and extreme (14%) relevance. Before the training, half of the respondents reported that they felt moderately prepared to discuss factors contributing to differences in disease incidence for gender/sex biases (52%), while the majority of remaining respondents felt slightly (19%) or not at all (19%) prepared.

The posttraining survey was completed by 21 faculty (52% response rate), of whom 15 (71%) had some preclerkship teaching responsibilities, 10 (48%) had some clerkship teaching responsibilities, and 18 (86%) did not identify as LGBTQ+. Normal Q-Q plot points for all five pre- and posttraining GSD comfort/awareness/knowledge-related questions fell on a 45-degree-angle reference line, so were deemed to follow a normal distribution. *F*-test values for these five questions (0.77, 0.59, 0.94, 1.13, and 1.03, respectively) were all below the *F* critical value of 2.23 ($\alpha = .05$, $df = 20$) and regarded as demonstrating similar variability. Following the training, a significantly increased number of the 21 respondents who completed both pre- and posttraining surveys self-reported they were very or extremely aware of the difference between gender and sex (38% and 33%, respectively; $p = .002$). A significantly increased number of respondents self-reported that gender was moderately, very, or extremely pertinent to their teaching materials (24%, 29%, and 29%, respectively), and no participants after the session indicated that gender had no relevance ($p = .04$). Furthermore, a significantly increased number of participants reported feeling moderately, very, or extremely prepared to teach on the factors underlying differences in disease incidence among different gender and sex subgroups (52%, 29%, and 10%, respectively; $p = .005$; Table 3).

A majority of respondents (81%) reported after the training that they probably or definitely learned something that would change

Copyright © 2024 Crosby et al. This is an open-access publication distributed under the terms of the Creative Commons Attribution license.

Table 3. Pre- and Posttraining Results (*N* = 21)

Question[a]	No. (%) Not at All	Slightly	Moderately	Very	Extremely	*M* (*SD*)	*p* (CI)	Power
How knowledgeable do you feel about the difference between sex and gender?								
Pretraining	0 (0)	3 (14)	10 (48)	7 (33)	1 (5)	3.3 (0.8)	.002 (−1.125 to −0.304)	.83
Posttraining	0 (0)	1 (5)	5 (24)	8 (38)	7 (33)	4.0 (0.9)		
How aware are you of health inequities that exist for transgender and/or nonbinary people?								
Pretraining	0 (0)	1 (5)	4 (19)	13 (62)	3 (14)	3.9 (0.7)	.80 (−0.441 to 0.346)	.80
Posttraining	0 (0)	2 (10)	4 (19)	9 (43)	6 (29)	3.4 (0.9)		
How relevant are the topics of sex (chromosomal, anatomical, hormonal, etc.) to your teaching materials for medical students?								
Pretraining	1 (5)	2 (10)	9 (43)	5 (24)	4 (19)	3.4 (1.1)	.17 (−0.588 to 0.112)	.80
Posttraining	1 (5)	2 (10)	5 (24)	8 (38)	5 (24)	3.7 (1.1)		
How relevant are the topics of gender (identity, expression, lived experience, etc.) to your teaching materials for medical students?								
Pretraining	1 (5)	6 (29)	4 (19)	7 (33)	3 (14)	3.2 (1.2)	.04 (−0.825 to −0.320)	.81
Posttraining	0 (0)	4 (19)	5 (24)	6 (29)	6 (29)	3.7 (1.1)		
How prepared do you feel to discuss the factors that contribute to differences in disease incidence for different genders and sexes?								
Pretraining	4 (19)	4 (19)	11 (52)	2 (10)	0 (0)	2.5 (0.9)	.005 (−1.341 to −0.278)	.83
Posttraining	1 (5)	1 (5)	11 (52)	6 (29)	2 (10)	3.3 (0.9)		

[a]Rated on a 5-point Likert scale (1 = *not at all*, 2 = *slightly*, 3 = *moderately,* 4 = *very,* 5 = *extremely*).

their teaching practice, and 95% indicated interest in attending similar training sessions in the future.

Participants' feedback comments on the session are shown in Table 4.

Discussion

Many published curricula and courses on topics related to diversity of gender or sexual orientation exist. Existing trainings address domains such as clinician understanding of GSD populations,[7] terminology,[8] and appropriate patient interactions.[9] All are important for helping providers strengthen the therapeutic alliance with their GSD patients and deliver thoughtful care. The faculty training described here contributes to these efforts as it explores the pervasiveness of cisnormative and binary framings around gender and sex in medical education and why this is problematic, as well as offering the opportunity to develop techniques to avoid these biases.

This training was educationally impactful to an audience that predominantly had already participated in previous training on GSD topics. Two of the three teaching domains that demonstrated significant improvement in reported comfort and awareness after the session reflected the major teaching points of the session, namely, the practice of a getting-to-the-root mindset and the avoidance of conflating gender and sex. Participants also reported a significant increase in awareness of the relevance of gender to their respective teaching content.

Open-ended feedback on the session (Table 4) noted the effectiveness of the small-group discussion, as illustrated by a comment that mentioned how the deconstruction of the provided national guidelines was "excellent for highlighting my and the medical community at large's blind spots to this issue." Literature on medical faculty development emphasizes the importance of training activities that facilitate reflection, interdisciplinary collaboration, and group discussion for the promotion of collegiality and creation of a learning community.[10] We incorporated all three of these items into a single 1-hour session, and it is encouraging to appreciate the benefits from the synthesis of these elements as demonstrated by the majority of respondents indicating an intent to change teaching practices and as supported by specific posttraining reflections (Table 4).

It is important to call attention to the nature of the learning environment for this training. One respondent commented on the importance of safe spaces for educators to discuss without the inhibitory fear of making a mistake. Though the classroom environment is typically intended to be an opportunity for trial-and-error for students, faculty are often not afforded similar leeway to make mistakes. Feedback on this session alluded to a need for space for faculty to openly discuss in the process of evaluating and adjusting their lecture content, as should be afforded to all learners.

Participant feedback also highlighted opportunities to improve the training. Only 15 minutes were allotted for the small-group discussion, and feedback indicated that additional time would have been helpful (Table 4). If the overall session were extended in length (e.g., 90 minutes rather than 60 minutes), the training would benefit from more time afforded to the small-group discussion. Other feedback indicated that more specific

Copyright © 2024 Crosby et al. This is an open-access publication distributed under the terms of the Creative Commons Attribution license.

Table 4. Posttraining Open-ended Feedback

Item	Feedback
Please explain your answer to the question: I learned something today that will change my teaching practice.	"I learned how to clearly and succinctly describe difference in incidence across genders and sexes, including naming when we are not clear on what is driving the differences in incidence."<br>"Opening the conversation with students about how to look at old sex/gendered data and what it might be based on."<br>"More overt commentary around where I do not have insight into the relationship between sex and incidence of a disease."<br>"Reminded me to always emphasize the basis for the sex-based differences, or the lack of knowledge about it."<br>"I will be going through my materials in my lectures and example cases to see if there are changes to be made to be more inclusive or specific to gender/sex definitions."<br>"I have a better understanding of how to present sex differences in physiology and disease."<br>"Greater sensitivity to gender dichotomy and will try to avoid it."<br>"Helpful to see more examples of language that needs to be changed and why."<br>"Catching self falling into ingrained habits gendering needlessly—choosing PERSON as the word. Anatomy-first language."<br>"I am now thinking about why and the root."<br>"I used to use terms for sex and gender interchangeably (such as male and man), something I will not do going forward."<br>"I have a much clearer understanding of the terminology and issues surrounding language used."<br>"Try to be thoughtful and inclusive—will continue to be and have students and us be curious."<br>"I'm more aware of the number of people who self identify as LGBTQIA+."<br>"How to frame conversations about using sex in disease prevalence."<br>"While informative, the session did not present anything new that I was previous not aware of."<br>"I was aware of the issue."<br>"Already expert on this."
Please describe any revisions that you intend to incorporate into your teaching style and/or lecture content as a result of this training.	"Making note of any research data/sources that split responses by gender and making note of shortcomings of it."<br>"I'm planning to change how I write the manuscript for a systematic review of biomarkers and HIV and HIV key population stigma."<br>"Thinking about what the cause of the differences noted are and not just assuming it is as simple as sex or gender."<br>"Relook at lectures for sex/gender bias."<br>"I will be thinking about this in rounds."<br>"Avoid using gender unless important to the case and if so, will try to explain the 'root' for its need."<br>"I will be more inclusive/less binary when describing people."<br>"I will be more careful with language to distinguish sex and gender."<br>"I plan to go through material and use 'patient' or 'person' whenever possible. As a module director I will ask faculty to do the same."<br>"Counseling all faculty to revise materials to be more sensitive to gender and sex."<br>"I intend to use more transparency when I discuss [United States Preventive Services Task Force] guidelines to students (ie this says to start mammograms at age 40 for women, when really we should be saying anyone with breasts, etc)."<br>"Will be cognizant of saying 'pregnant person' when discussing pregnancy."<br>"See previous item."<br>"None."
Please describe any areas of continued confusion.	"There are many areas where we don't know the science for WHY there are sex differences for diseases. Nevertheless, these issues will cause disruption to the learning environment. What are phrases to use to help the learning group get back on track?"<br>"How do you create content that is inclusive without being overwhelming? The concept that improving patient care is very important, however too much detailed information may be overwhelming. I wanted more specific examples of simplifying teaching while still being inclusive."<br>"Had moved to being clear about sex v gender and beyond the binary but presenting data that is inadequate is still an adjustment in terms of knowing how deep to go each time."<br>"Causation in disease is such a complex process and likely multi-factorial. There will be elements that are behavioral/societal, dietary, related to genetic ancestry, size/body weight as well as related to hormones—while I understand the importance of separating 'gender' is it still acceptable to say there is a higher prevalence in patients of female sex if accompanied by the caveat that one is uncertain of whether the causality is genetic, hormonal, average size/body fat, etc. and thereby highlighting that there may be a complex interplay in the relationship?"<br>"Would love to know more about what it is about certain diseases that make them more prevalent in one sex so that we can more accurately teach this (for example, autoimmune diseases in females—how can we frame this to students? Is it estrogen levels? I realize it would be hard to do this for every disease process)."<br>"I think I have a broad understanding now, but need to work more on more nuanced areas."<br>"Not right now but hope there is someone/place to continue to talk about this safely as an educator. Please keep having these—you only touched a small portion of the faculty and this is important stuff."<br>"None."<br>"None."

(Table continues)

Copyright © 2024 Crosby et al. This is an open-access publication distributed under the terms of the Creative Commons Attribution license.

Table 4. Continued

Item	Feedback
Please describe any aspects of this workshop session that you found particularly helpful or effective and any aspects that you thought were lacking or needed improvement.	"The case and discussion questions were especially effective and memorable for consolidating what we learned. Thought the environment was welcoming and allowed for people to express misunderstandings (for example, about gender/sex as a biological vs. social construct), while still addressing the misunderstanding. Perhaps we covered this, but I don't remember discussing structural inequities and inequities in disease outcomes for gender and sexually diverse people. As a parenthetical note, the case did a great job of describing 'alcohol use disorder."' "Bringing up aspects we need to consider and provided food for thought. It is good that the emphasis is that the answers are very nuanced. But the recommendations at the end leave the (semi-trained) instructor to come up with ways of addressing the issues. While specific guidelines are impossible, in some cases, recommendations could have been made more specific. Also, the presentation could have touched on the disparity in terminology in the target population (both students and patients). While the students may get training on the proper terminology, the patients do not. Therefore, it is always a good idea to define terms in advance, which can also incorporate stating the limitations when using them as shortcuts. I think it may be good to emphasize more that in some cases, using terms and shortcuts is unavoidable, and ok to do, as long as it is kept to a minimum and accompanied by a disclaimer. Otherwise people may get confused or worry too much about details. Students often do not realize that no term is unbiased and all-inclusive. For example, most know the limitations of male/female, but some believe that XX/XY are unambiguous." "The guidelines review was excellent for highlighting my and the medical community at large's blind spots to this issue. Very powerful." "Great to split into the groups to brainstorm with student and faculty facilitating. Can't think of something to improve—great job." "I particularly liked the exercise—it's fascinating that guidelines that are intended to cover everyone exclude and alienate so many people." "The case practice." "I liked the breakout group part the most—would love if it even went a step further to force us to actually rewrite guidelines!" "Would have been nice to have a debrief after the small groups." "The timeline and data about sexual identity was helpful." "Eye-opening to see the statistics shared in the beginning, and super helpful to see examples of addressing ways we need to change labels and language in medicine. More comparison of incorrect vs corrected language would be helpful and a reference faculty who did not attend this session can go to online while preparing lectures and notes would be great!" "How do we get to all of the people teaching students effectively? Setting, size of group and timing made it challenging to go into the case example too far. Would have been great to have several and smaller groups—more time would have been helpful."

recommendations for the implementation of a getting-to-the-root mindset would have been appreciated. This comment is mirrored by the finding that, though a statistically significant number of respondents indicated increased preparedness to utilize a getting-to-the-root approach after the session, the majority still felt only moderately prepared (Table 3). Ideally, additional sessions, variety and complexity of case examples, and opportunities for practice would better support faculty preparation.

While the content of this training session has been designed for both undergraduate and graduate medical educators, we only surveyed participants' roles in the undergraduate medical curriculum. Future adaptations of this training session could consider specific outreach to graduate medical educators to understand its impact at later phases of medical education.

The overall findings from the session are particularly compelling as there is a paucity of prior literature on medical faculty training in teaching on gender and sex in a manner inclusive of GSD health. Given the supportive feedback and various strengths delineated in the survey results, the structure of the training session is effective and flexible and can be utilized to guide future training both within and beyond our institution. The training is also effective in that it is relatively short and inexpensive, as the session has been designed to last for a single lunch hour to accommodate audience availability.

There are important limitations to acknowledge regarding our interpretation of this training session and its impact. Only 36 participants completed the pretraining survey, and because many participants were unable to stay through the session due to professional responsibilities, only 21 posttraining survey responses were collected. Thus, despite reported power values of above 80% in all five questions, the small sample size still inherently limits the statistical significance of our results and our assuredness of the session's potential impact at other institutions. Furthermore, we were unable to rule out chance or to complete a sensitivity analysis of our results. A potential selection bias

Copyright © 2024 Crosby et al. This is an open-access publication distributed under the terms of the Creative Commons Attribution license.

underlies the survey results as faculty self-selected to attend this optional training session and presumably had inherent interest in learning about GSD-related topics, so our results may not be wholly representative of overall faculty consensus. Lastly, pre- and posttraining survey questions were based on self-reported perceptions of knowledge, comfort, and awareness and did not objectively test respondent skills or knowledge.

This 1-hour training session introduces and guides medical educators to thoughtfully teach about gender and sex in a manner that is accurate and more inclusive of GSD health. Similar forums in the future with particular attention to case presentation examples on utilizing a getting-to-the-root approach to gender and sex will be helpful to continue supporting faculty learning.

Appendices

A. Key Terms.docx

B. Presentation With Speaker Notes.pptx

C. Small-Group Discussion Questions.docx

D. Facilitator Guide.docx

E. Handout Form (Printable Version, Trifold Format).pdf

F. Handout Form (Electronic Version, Standard Format).pdf

G. Pre- and Posttraining Survey Forms.docx

All appendices are peer reviewed as integral parts of the Original Publication.

Benjamin Crosby: Third-Year Medical Student, Boston University Chobanian & Avedisian School of Medicine; ORCID: https://orcid.org/0000-0002-6001-707X

Hannah Dumas, MPH: Third-Year Medical Student, Boston University Chobanian & Avedisian School of Medicine

Janet Monroe: Fourth-Year Medical Student, Boston University Chobanian & Avedisian School of Medicine

Fredric Fabiano, MS: Second-Year Medical Student, Boston University Chobanian & Avedisian School of Medicine

Isabelle Gell-Levey: Fourth-Year Medical Student, Boston University Chobanian & Avedisian School of Medicine

Christopher Noyes: Fourth-Year Medical Student, Boston University Chobanian & Avedisian School of Medicine

Kikuye Sugiyama: Third-Year Medical Student, Boston University Chobanian & Avedisian School of Medicine

Jennifer Siegel, MD: Medical Director, Transgender Health Program, Massachusetts General Hospital

Angelique Harris, PhD: Associate Dean, Office of Diversity and Inclusion, Boston University Chobanian & Avedisian School of Medicine; Associate Professor, Department of General Internal Medicine, Boston Medical Center

Carl Streed Jr, MD, MPH: Research Lead, GenderCare Center, Boston Medical Center; Assistant Professor, Section of General Internal Medicine, Boston University Chobanian & Avedisian School of Medicine

Ann C. Zumwalt, PhD: Associate Professor, Department of Anatomy and Neurobiology, Boston University Chobanian & Avedisian School of Medicine

Acknowledgments
We wish to acknowledge the generous financial support received from the Massachusetts Medical Society's 2022 LGBTQ Health Disparities Grant. We would like to acknowledge our consultant, Jessica Halem, for her invaluable support in providing expert guidance during the development of the project and for presenting on the history of gender and sexually diverse health care during the session.

Disclosures
None to report.

Funding/Support
Benjamin Crosby received funding from the Massachusetts Medical Society 2022 LGBTQ Health Disparities Grant.
Dr. Carl Streed reports salary support from a National Heart, Lung, and Blood Institute career development grant (1K01HL151902), an American Heart Association career development grant (20CDA35320148), the Doris Duke Charitable Foundation (2022061), and the Boston University Chobanian & Avedisian School of Medicine Department of Medicine Career Investment Award.

Prior Presentations
Crosby B, Dumas H, Monroe J, et al. Faculty training on gender & sex teachings. Poster presented at: 17th Annual McMahan Education Conference; May 25, 2022; Boston, MA.

Ethical Approval
The Boston University Medical Campus Institutional Review Board deemed further review of this project not necessary.

Disclaimer
The views expressed in this article are the authors' own and are not an official position of our primary teaching institute, Boston University Chobanian & Avedisian School of Medicine, or our funder, the Massachusetts Medical Society.

References

1. Safer JD, Coleman E, Feldman J, et al. Barriers to healthcare for transgender individuals. *Curr Opin Endocrinol Diabetes Obes*. 2016;23(2):168-171. https://doi.org/10.1097/MED.0000000000000227

Copyright © 2024 Crosby et al. This is an open-access publication distributed under the terms of the Creative Commons Attribution license.

2. Streed CG Jr, Beach LB, Caceres BA, et al; American Heart Association Council on Peripheral Vascular Disease; Council on Arteriosclerosis, Thrombosis and Vascular Biology; Council on Cardiovascular and Stroke Nursing; Council on Cardiovascular Radiology and Intervention; Council on Hypertension; and Stroke Council. Assessing and addressing cardiovascular health in people who are transgender and gender diverse: a scientific statement from the American Heart Association. *Circulation*. 2021;144(6):e136-e148. https://doi.org/10.1161/CIR.0000000000001003

3. Bradford J, Reisner SL, Honnold JA, Xavier J. Experiences of transgender-related discrimination and implications for health: results from the Virginia Transgender Health Initiative Study. *Am J Public Health*. 2013;103(10):1820-1829. https://doi.org/10.2105/AJPH.2012.300796

4. Safer JD, Tangpricha V. Care of the transgender patient. *Ann Intern Med*. 2019;171(1):ITC1-ITC16. https://doi.org/10.7326/AITC201907020

5. Osmosis from Elsevier. Sexual orientation and gender identity. *YouTube*. June 18, 2020. Accessed July 12, 2024. https://www.youtube.com/watch?v=xCMmZUu07IQ

6. Drinking levels defined. National Institute on Alcohol Abuse and Alcoholism. Updated 2023. Accessed July 12, 2024. https://www.niaaa.nih.gov/alcohol-health/overview-alcohol-consumption/moderate-binge-drinking

7. Gallego J, Knudsen J. LGBTQI' defined: an introduction to understanding and caring for the queer community. *MedEdPORTAL*. 2015;11:10189. https://doi.org/10.15766/mep_2374-8265.10189

8. Ulrich IP, Harless C, Seamon G, et al. Implementation of transgender/gender nonbinary care in a family medicine teaching practice. *J Am Board Fam Med*. 2022;35(2):235-243. https://doi.org/10.3122/jabfm.2022.02.210182

9. Paul CR, Wolfe AD, Catallozzi M, Jirasevijinda T, Kutscher E, Lurie B. Teaching sexual orientation and gender identity in pediatric clinical settings: a training workshop for faculty and residents. *MedEdPORTAL*. 2021;17:11137. https://doi.org/10.15766/mep_2374-8265.11137

10. McLean M, Cilliers F, Van Wyk JM. Faculty development: yesterday, today and tomorrow. *Med Teach*. 2008;30(6):555-584. https://doi.org/10.1080/01421590802109834

Received: September 24, 2023
Accepted: April 1, 2024
Published: August 13, 2024

Copyright © 2024 Crosby et al. This is an open-access publication distributed under the terms of the Creative Commons Attribution license.
